# Prefoldins are novel regulators of the unfolded protein response in artemisinin resistant *Plasmodium falciparum* malaria

**DOI:** 10.1016/j.jbc.2024.107496

**Published:** 2024-06-24

**Authors:** Rumaisha Shoaib, Nidha Parveen, Vikash Kumar, Ankita Behl, Swati Garg, Preeti Chaudhary, Devasahayam Arokia Balaya Rex, Monika Saini, Preeti Maurya, Ravi Jain, Kailash C. Pandey, Mohammad Abid, Shailja Singh

**Affiliations:** 1 Special Centre for Molecular Medicine, Jawaharlal Nehru University, New Delhi, Delhi, India; 2 Medicinal Chemistry Laboratory, Faculty of Life Sciences, Department of Biosciences, Jamia Millia Islamia, New Delhi, Delhi, India; 3 Parasite Host Biology Group, ICMR-National Institute of Malaria Research, New Delhi, India; 4 Department of Life Sciences, IGNOU, Delhi, India; 5 Department of Laboratory Medicine and Pathology, Mayo Clinic, Rochester, Minnesota, USA; 6 Department of Life Sciences, Shiv Nadar University, Delhi, Uttar Pradesh, India

**Keywords:** malaria, *Plasmodium falciparum*, prefoldin, artemisinin resistance, MSP-1, Biperiden, α-tubulin-I

## Abstract

Emerging Artemisinin (ART) resistance in *Plasmodium falciparum* (*Pf*) poses challenges for the discovery of novel drugs to tackle ART-resistant parasites. Concentrated efforts toward the ART resistance mechanism indicated a strong molecular link of ART resistance with upregulated expression of unfolded protein response pathways involving Prefoldins (PFDs). However, a complete characterization of PFDs as molecular players taking part in ART resistance mechanism, and discovery of small molecule inhibitors to block this process have not been identified to date. Here, we functionally characterized all *Pf* Prefoldin subunits (PFD1-6) and established a causative role played by PFDs in ART resistance by demonstrating their expression in intra-erythrocytic parasites along with their interactions with Kelch13 protein through immunoprecipitation coupled MS/MS analysis. Systematic biophysical interaction analysis between all subunits of PFDs revealed their potential to form a complex. The role of PFDs in ART resistance was confirmed in orthologous yeast PFD6 mutants, where *Pf*PFD6 expression in yeast mutants reverted phenotype to ART resistance. We identified an FDA-approved drug “Biperiden” that restricts the formation of Prefoldin complex and inhibits its interaction with its key parasite protein substrates, MSP-1 and α-tubulin-I. Moreover, Biperiden treatment inhibits the parasite growth in ART-sensitive *Pf*3D7 and resistant *Pf*3D7k13^R539T^ strains. Ring survival assays that are clinically relevant to analyze ART resistance in *Pf*3D7k13^R539T^ parasites demonstrate the potency of BPD to inhibit the growth of survivor parasites. Overall, our study provides the first evidence of the role of *Pf*PFDs in ART resistance mechanisms and opens new avenues for the management of resistant parasites.

Malaria is one of the biggest threats to global health, which has existed alongside people for more than 40 centuries. Artemisinin-based combination therapies (ACTs) are the first line of recommended treatment for the malaria burden. However, resistance to artemisinin and ACTs in *Plasmodium falciparum* is spreading rapidly throughout Southeast Asia, posing a threat to control worldwide malaria eradication and elimination (1). Several mechanisms are proposed to play a role in the development of artemisinin resistance in the *Plasmodium* parasite. Clinical artemisinin resistance is strongly linked to polymorphism (Y493H, R539T, I543T, and C580Y) in the β-propeller domain of the Kelch13 (K13) protein (PF3D7_1343700) (2). This specific domain is entailed to bind with interacting substrates and allow them for ubiquitination. The function of Kelch13 is not well understood in *Plasmodium*, however, it shows similarity to Kelch/BTB/POZ ubiquitination adapters. K13 is hypothesized to function as a ubiquitin-ligase adapter and targets the ubiquitin-mediated degradation of proteins (3, 4).

A recent study carried out *in vivo* transcriptomic analysis of 1048 *P. falciparum* isolates from patients with acute malaria and reported that artemisinin resistance is related to enhanced expression of unfolded protein response pathways involving the main *Plasmodium* reactive oxidative stress complex and Tailless complex polypeptide 1 Ring Complex (TRiC) chaperone complexes. The same report investigated the expression profile of genes linked to artemisinin resistance and found overexpression of one of the genes “Prefoldin” in the artemisinin-resistant patient sample (5). Another report on the *Leishmania* parasite revealed that FAZP (Prefoldin homolog of *Plasmodium*) is overexpressed by 3.5-fold in ART-resistant parasites in comparison to wild-type parasites (6). In light of the above facts, we selected Prefoldin (PFD) family of proteins to explore their functions in malaria parasite and further attempted to investigate their relevance with respect to artemisinin resistance. Prefoldin is an evolutionarily conserved hetero-hexameric molecular co-chaperone, vital for maintaining cellular homeostasis in both physiological and pathological conditions. PFD complexes are ubiquitously present in all archaea and eukaryotes, and their main function is to capture nascent polypeptides and deliver them to Chaperonin Containing Tailless complex polypeptide 1 (CCT) or TRiC for proper folding. As of now, mainly Prefoldin’s roles have been identified in the folding of cytoskeletal proteins actin and tubulin. However, our present understanding of Prefoldin's possible function in protein homeostasis maintenance is restricted. Surprisingly, each of the single subunits can bind to independent unique interactors, allowing them to execute diverse tasks (7).

Since the emergence and spread of parasites resistant to ACTs have made the strategies more difficult to control the disease, a comprehensive understanding of parasite factors that govern the success of the pathogen in making resistant to current antimalarials is strongly required for therapeutic intervention and disease management. In line with this, lack of understanding of the PFD complex in the *Plasmodium* parasite represents a significant void in our functional understanding of chaperones and co-chaperones and demands to explore their role in relevance to artemisinin resistance. In the present study, we delineated the role of Prefoldins in the artemisinin-mediated resistance mechanism by demonstrating its interaction with the K13 protein in ART-sensitive *Pf*3D7 and resistant *Pf*3D7k13^R539T^ strains. We identified and characterized the orchestrated assembly of the PFD co-chaperone heteroprotein complex in *P. falciparum* and elucidated its functional significance in the unerring folding of crucial cytoskeletal and invasion-related parasite proteins, specifically *Pf*α-tubulin-I and Merozoite Surface Protein (MSP-1), respectively. Through an examination of FDA-approved chemotypes targeting *Pf*PFD, this study unveils 1-{bicyclo[2.2.1]hept-5-en-2-yl}-1-phenyl-3-(piperidin-1-yl)propan-1-ol (commonly referred to as Biperiden or BPD) as a potential inhibitor of the *Pf*PFD complex, exhibiting substantial anti-plasmodial efficacy. The functional complementation assay conducted in the orthologous system *Saccharomyces cerevisiae* demonstrates rescued yeast growth and affirms the reduced sensitivity of artemisinin in the PFD6 complemented strain. Using a clinically relevant ring survival assay, we demonstrate that *Pf*PFDs inhibitor BPD reduces the number of parasite survivors following artemisinin treatment in resistant parasites. Overall, our study contributes to understanding the role of *Pf*PFDs in providing artemisinin resistance and present BPD as a promising candidate for further exploration as an antimalarial agent. These findings offer a unique opportunity to tackle artemisinin resistance management and the development of novel antimalarial chemotherapeutics.

## Results

### Prefoldins identified as interacting partners of *Pf*Kelch13

**Figure 1:**
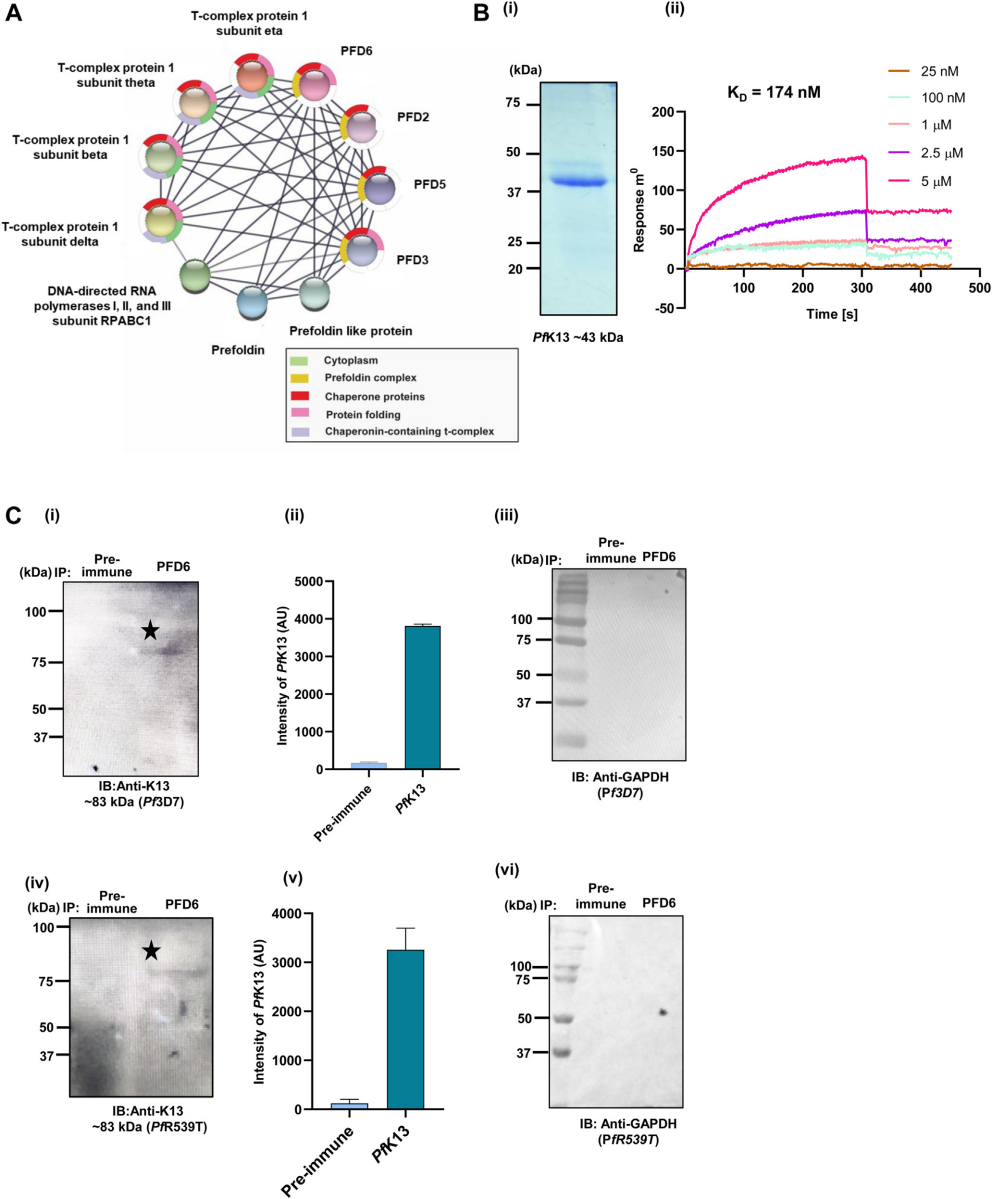
**Interaction studies of Prefoldin subunits with *PfK13*.***A*, protein–protein interaction network generated using STRING showing the interaction of *PfK13* with Prefoldin subunits. *B*, (i) *Overexpression and purification of PfK13.* Coomassie-stained gel showing the purified recombinant protein *Pf*K13 (∼43 kDa). (ii) *Evaluation of the interaction strength between Pf*K13 and *Pf*PFD6. SPR-based interaction analysis upon immobilizing *Pf*K13 and titrating *Pf*PFD6 displayed a good binding strength with a K_D_ value of 174 nM. Data were fit to the two-state conformational change model using AutoLab SPR Kinetic Evaluation software. K_D_ value was calculated using the Integrated Rate Law (IRL) equation as described in experimental procedures. *C*, *co-immunoprecipitation assays validating the binding of PfPFD6 with PfK-13.* Anti-*Pf*PFD6 antisera was cross-linked to amino coupling plus resin followed by incubation with *P. falciparum* 3D7 (i) and *Pf*3D7k13^R539T^ lysate (iv). Eluted fractions were resolved on 12% SDS-PAGE and subjected to Western blotting using anti-*Pf*K13 (1:1000) antibodies and their respective HRP-conjugated anti-rat antibodies (1:5000). Immunoprecipitation assays were performed from two independent samples prepared from two independent experiments, and a representative blot is shown. (ii, v) Intensity plots of bands obtained for *Pf*K13 in co-immunoprecipitation assays depicted in i and iv using ImageJ. AU depicts arbitory units. Error bars represent mean ± standard deviation among two independent biological replicates. (iii, vi) Eluted fractions of co-immunoprecipitation assays illustrated in i, iv were subjected to Western blotting using anti-GAPDH antibodies.

A previous study reported that artemisinin resistance is linked with up-regulated expression of unfolded protein response pathways involving Prefoldins of malaria parasite (5). Also, artemisinin resistance is strongly associated with the polymorphism in the β-propeller domain of the K13 protein. In light of the abovementioned facts, we performed Co-immunoprecipitation (Co-IP) studies where an anti-K13 antibody was crosslinked to AminoLink plus Coupling Resin to pull down the interacting partners from parasite lysate. The eluted fractions were subjected to mass spectrometry analysis. The hits obtained are provided in Fig. S1 and were used to generate protein–protein interaction network using STRING with special emphasis given to chaperones and chaperonin containing T complex. These data clearly demonstrate the possibility of interaction between *Pf*PFDs with *Pf*K13 (Fig. 1*A*).

**Figure 2:**
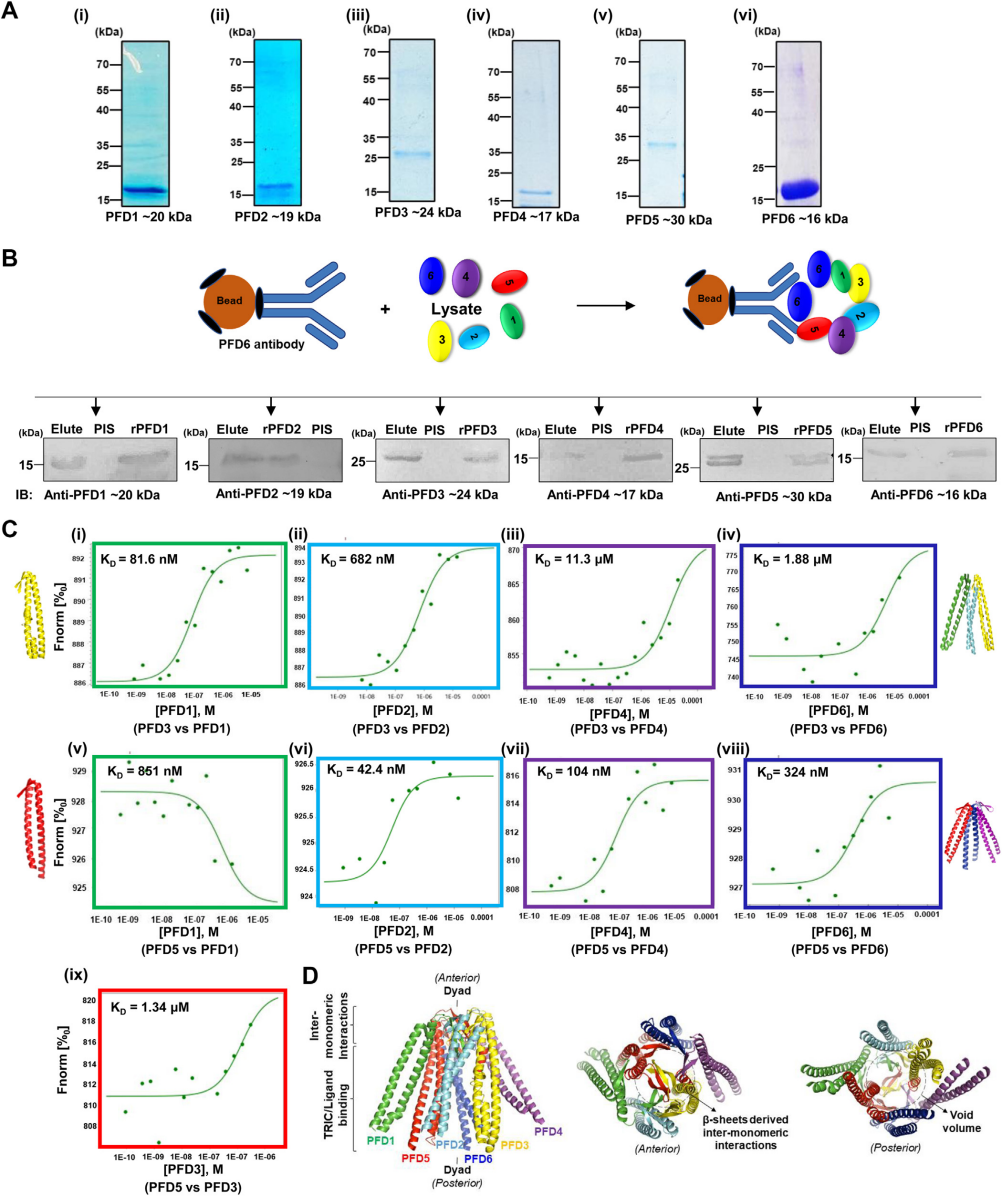
**Assembly of *Pf*PFDs to form a complex.***A*, *SDS-PAGE of purified recombinant prefoldin subunits. Pf*PFD (20 kDa), *Pf*PFD (19 kDa), *Pf*PFD3 (24 kDa), *Pf*PFD4 (17 kDa), *Pf*PFD5 (30 kDa), *Pf*PFD6 (16 kDa). *B*, *co-immunoprecipitation assay showing PfPFD complex formation.* Anti-*Pf*PFD6 antiserum was cross-linked to amino coupling plus resin followed by incubation with parasite lysate. Eluted fractions were resolved on 12% SDS-PAGE and subjected to Western blotting using in-house generated *Pf*PFD1-6 antisera (1:1000 of each) and their respective HRP-conjugated anti-mice antibodies (1:5000). PIS represents samples crosslinked with preimmune sera. *C*, *microscale thermophoresis for complex formation analysis. Pf*PFD3 (i, ii, iii, iv) and *Pf*PFD5 (v, vi, vii, viii) (20 μM of each) were labelled using RED-NHS dye and titrated with increasing concentrations of other *Pf*PFD subunits. Binding affinity (K_D_) as determined by steady-state analysis, are as follows: (i) 81.6 nM (*Pf*PFD3-*Pf*PFD1), (ii) 682 nM (*Pf*PFD3-*Pf*PFD2), (iii) 11.3 μM (*Pf*PFD3-*Pf*PFD4), (iv) 1.88 μM (*Pf*PFD3-*Pf*PFD6) indicating higher binding affinity of *Pf*PFD3 with *Pf*PFD1 and *Pf*PFD2. Similarly, K_D_ values of (v) 851 nM (*Pf*PFD5-*Pf*PFD1), (vi) 42.4 nM (*Pf*PFD5-*Pf*PFD2), (vii) 104 nM (*Pf*PFD5-*Pf*PFD4), and (viii) 324 nM (*Pf*PFD5-*Pf*PFD6) and (ix) 1.34 μM (*Pf*PFD5-*Pf*PFD3), were observed indicating higher binding affinity of *Pf*PFD5 with *Pf*PFD4, *Pf*PFD4 and *Pf*PFD6. *D*, *overall architecture of the PfPFD*_*hexamer*_. Structural models of *Pf*PFD1-6 were generated using I-TASSER, and *Pf*PFD_hexamer_ structure was generated using human TRiC-PFD complex (PDB ID: 6NR8) as a template. Upon rigid-body superimposition of both the complexes, the overall RMSD value of the C-alpha atomic co-ordinates was found to be 1.45 Å, suggesting a reliable 3D structure to be taken further for *in silico* and *in vitro* interaction analysis, and inhibition studies. *Pf*PFD_hexamer_ structural model revealed potential of *Pf*PFDs to form a hetero-hexamer with a jellyfish-like architecture.

To confirm the direct interaction between prefoldins and *Pf*K13, we performed an SPR-based interaction analysis. *Pf*K13 was purified using Nickel NTA based affinity chromatography (Fig. 1*B*i) and recombinant *Pf*PFD6 and *Pf*K13 were used to quantify the interaction strength by SPR. Here immobilized *Pf*PFD6 was titrated with increasing concentrations of *Pf*K13. A K_D_ value of 174 nM was observed for *Pf*PFD6 - *Pf*K13 interaction (Fig. 1*B* ii). The interaction of *Pf*PFD6 with *Pf*K13 was further validated using co-immunoprecipitation assays in *Pf*3D7 and artemisinin-resistant strain *Pf*3D7k13^R539T^. Here, *Pf*PFD6 antiserum was cross-linked to the AminoLink plus Coupling Resin, followed by incubation with the parasite lysate prepared from the mix-stage parasite population. Bound protein fractions were eluted and subjected to western blotting by probing with anti-*Pf*K13 and GAPDH antibodies. The desired protein band of *Pf*K13 was observed in the eluted fractions post-probing with anti-*Pf*K13 antibodies (Fig. 1*C* i). These results suggest the interaction of *Pf*PFD6 with *Pf*K13 in *Pf*3D7. Interestingly, the desired protein band of *Pf*K13 was observed in *Pf*3D7k13^R539T^, further suggesting the interaction of both proteins in artemisinin-resistant strain (Fig. 1*C* iv). Intensity plots of *Pf*K13 bands obtained are represented in Figure 1*C* ii, v. Blot probed with anti-GAPDH antibodies showed no band (Fig. 1*C* iii, vi) which suggest specific interaction pair of *Pf*PFD6 are being pulled down in the assay. Additionally, Figure 2*B* reveals that anti-*Pf*PFD6 antibody pulls the PFD complex as a band of each *Pf*PFD protein was detected when probed with their specific antisera. However, no band was observed with pre-immune sera, further suggesting the specificity of *Pf*PFD6 antibody towards pulling the specific interaction pairs of *Pf*PFD6.

### *Pf*PFD1-6 expressed during asexual blood stages in *Pf*3D7 and artemisinin-resistant line *Pf*3D7k13^R539T^

**Figure 3:**
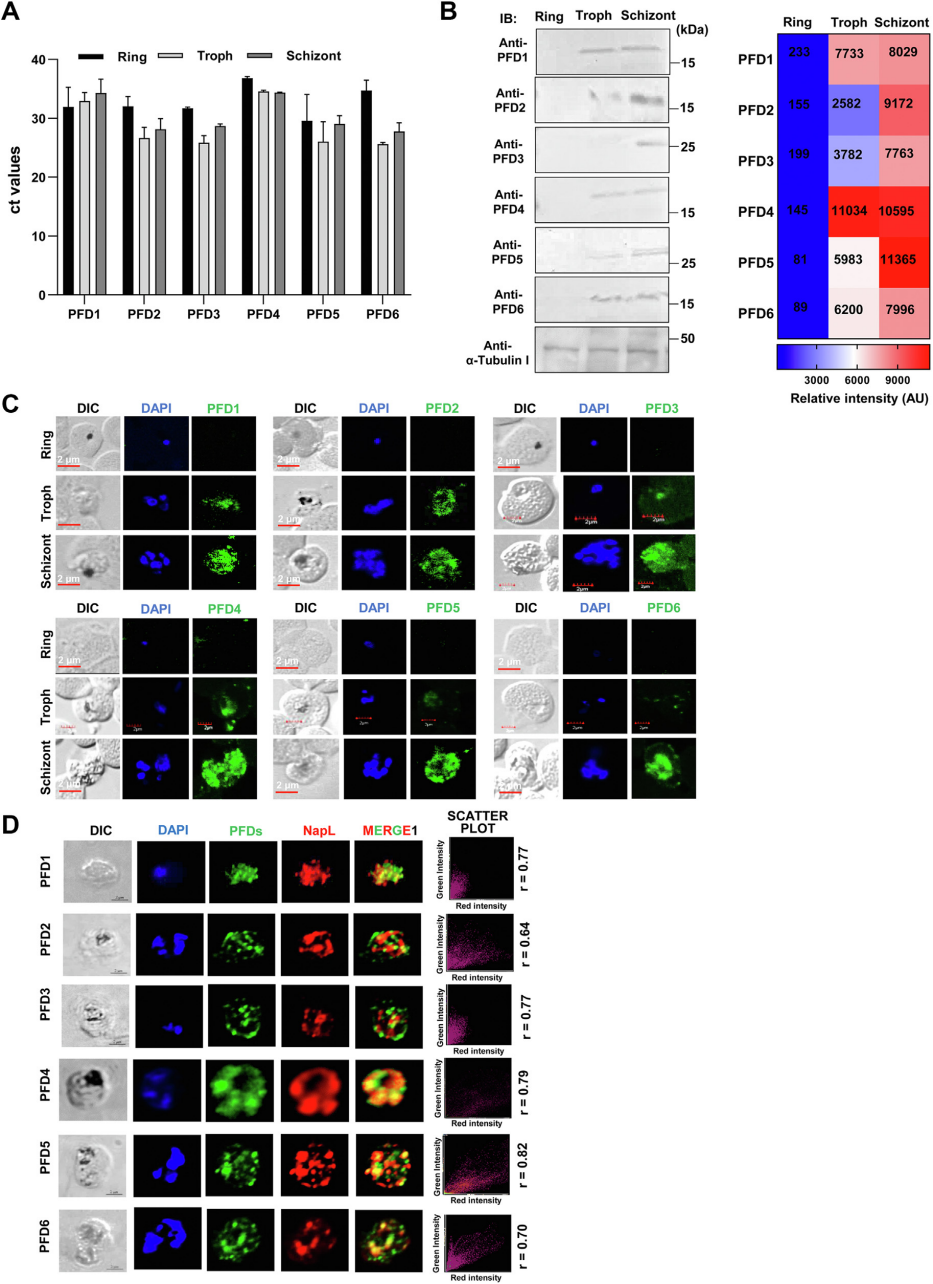
**Expression and localization of *Pf*PFDs in asexual blood stages of *P. falciparum*.***A*, *RT-qPCR analysis.* Bar graph plot representing Ct-values of *Pf*PFD1-6 in different asexual stages of the parasite. Error bars represent values of mean ± standard deviation among independent measurements from three biological repeats done in duplicates. *B*, *stage-specific expression of PfPFD1-6 at protein levels.* (i) Parasite lysates were prepared for different asexual stages of the parasite and subjected to western blotting. Immunoblotting with *Pf*PFD1-6 antisera (1:1000 of each) and HRP- conjugated anti-mice antibodies (1:5000) showed differential expression of *Pf*PFD1-6 across different asexual stages of the parasite. (ii) Heat map shows differential expression of *Pf*PFD at different intraerythrocytic stage. Shading of blue and red depict lower and higher expression levels, respectively. *C*, *cellular localization of PfPFD1-6*. Smears of methanol-fixed *Pf*3D7-infected erythrocytes were stained with anti-*Pf*PFD1-6 antibodies (1:200) followed by incubation with Alexa Fluor-conjugated secondary antibodies (Alexa Fluor 488, *green*). DIC: differential interference contrast image, DAPI: nuclear staining 40, 6-diamidino-2-phenylindole (*blue*); *Pf*PFD1-6: mouse anti-*Pf*PFD1-6 (*green*) (Scale bar: 2 μm). IFA depicted that *Pf*PFD1-6 are expressed at the trophozoite and schizont stage of the parasite. *D*, *PfPFD1-6 are mostly confined to the cytoplasm of schizonts*, as confirmed by its co-localization with a cytosolic protein marker, *Pf*NapL. DIC: differential interference contrast image, DAPI: nuclear staining 40, 6-diamidino-2-phenylindole (*blue*); *Pf*PFD1-6: mouse anti- *Pf*PFD1-6 (*green*); *Pf*NapL: anti-*Pf*NapL antibody (*red*); merge 1: overlay of *Pf*PFD1-6 with *Pf*NapL; merge 2: overlay of DAPI, *Pf*PFD1-6 and *Pf*NapL (Scale bar: 2 μm).

Reverse Transcriptase-quantitative Polymerase Chain Reaction (RT-qPCR) stands as a precise and convenient technique for quantifying target gene expression levels. RT-qPCR revealed that the transcripts encoding for *Pf*PFD1-6 are expressed throughout the intra-erythrocytic life cycle of *P. falciparum* (Fig. 3*A*). As a positive control, transcripts encoding for 18S rRNA were amplified using *Pf18s*-specific primers.

Western blot analysis of the native *Pf*PFD1-6 in the parasite lysate prepared from all three intra-erythrocytic stages corroborated the expression of *Pf*PFD1-6 in the parasite (Fig. 3*B*). At the trophozoite and schizont stages, distinct bands of *Pf*PFD1-6 were detected with their anticipated molecular weights (18.4 kDa: *Pf*PFD1, 16.6 kDa: *Pf*PFD2, 22.6 kDa: *Pf*PFD3, 15.3 kDa: *Pf*PFD4, 29.1 kDa: *Pf*PFD5, and 13.6 kDa *Pf*PFD6). However, at the ring stage, the expression of *Pf*PFD1-6 was not detected. *Pf*αTubulin-I served as a loading control (Fig. 3*B*). Full uncropped blots of these results are represented in Fig. S2. Furthermore, expression of PFDs was not detected in the *E. coli* lysate or uninfected RBCs (both insoluble and soluble fractions) which served as negative controls (Fig. S3). We also tested the expression of *Pf*PFD1-6 in artemisinin-resistant line *Pf*3D7k13^R539T^ and found that Prefoldins expression is upregulated in *Pf*3D7k13^R539T^ as compared to artemisinin-sensitive *Pf*3D7 (Fig. S4). RT-PCR and Western-based expression analysis were validated by Immunofluorescence Assays (IFAs), which depicted that *Pf*PFD1-6 are expressed at the trophozoite and schizont stages of the parasite (Fig. 3*C*). Altogether, these findings suggest that *Pf*PFD1-6 is expressed at both the transcript and protein levels in the intra-erythrocytic stages of the parasite. Furthermore, *Pf*PFD1-6 is mostly confined to the cytoplasm of schizonts, as confirmed by its co-localization with a cytosolic protein marker, *Pf*NapL (Fig. 3*D*).

### *Pf*PFD_s_ form a hetero-hexameric complex

CDS for *pfpfd1, pfpfd2, pfpfd3, pfpfd4, pfpfd5,* and *pfpfd6* were cloned in pET-28a(+), over-expressed, and recombinant *Pf*PFD subunits (6x-His-*Pf*PFD1-6; or, r*Pf*PFD1-6) were purified. r*Pf*PFD1-6 resolved on SDS-PAGE as a protein species of 20, 19, 24, 17, 30, and 16 kDa, respectively (Fig. 2*A*). Polyclonal antibodies were raised against r*Pf*PFD1-6 in male BALB/c mice. *Pf*PFD complexation was investigated using Co-IP, in which *Pf*PFD6 antiserum was cross-linked to AminoLink plus Coupling Resin followed by incubation with the parasite lysate. Eluted fractions were resolved on SDS-PAGE, followed by western blotting with the respective *Pf*PFD1-6 antisera. Each blot showed a respective band of the *Pf*PFD subunits (Fig. 3*B*), suggesting that *Pf*PFD1-6 forms a complex similar to their counterparts in other species. Full uncropped blots are represented in Fig. S2.

In archaea and eukaryotes, PFD2 and PFD6 interact with PFD3 and PFD5, respectively, to generate sub-complexes. This is followed by the recruitment of PFD1 and PFD4, which are then assembled by these sub-complexes to form a complex (8). In light of the above facts, we attempted to identify how PFD subunits in *P. falciparum* assemble to form a complex. We performed an MST analysis to investigate the interaction of *Pf*PFD3 and *Pf*PFD5 with other subunits. Here labelled *Pf*PFD3 and *Pf*PFD5 were titrated with decreasing concentrations of other *Pf*PFD subunits. Figure 2*C* shows the dose–response curve and MST signal for *Pf*PFD3 and *Pf*PFD5 binding with other subunits. Binding affinity (K_D_) values obtained are as follows: 81.6 nM (*Pf*PFD3-*Pf*PFD1), 682 nM (*Pf*PFD3-*Pf*PFD2), 11.3 μM (*Pf*PFD3-*Pf*PFD4), and 1.88 μM (*Pf*PFD3-*Pf*PFD6), 851 nM (*Pf*PFD5-*Pf*PFD1), 42.4 nM (*Pf*PFD5-*Pf*PFD2), 1.34 μM (*Pf*PFD5-*Pf*PFD3), 104 nM (*Pf*PFD5-*Pf*PFD4), and 324 nM (*Pf*PFD5-*Pf*PFD6). Based on binding affinity values, *Pf*PFD3 was found to have higher binding affinity for *Pf*PFD1 and *Pf*PFD2 as compared to *Pf*PFD4 and *Pf*PFD6. Also, *Pf*PFD5 shows more binding affinity for *Pf*PFD2 and *Pf*PFD4 and *Pf*PFD6 as compared to *Pf*PFD1 and *Pf*PFD3. These data suggest that *Pf*PFDs can interact with each other to form a complex.

We next attempted to generate a reliable structural model of the *Pf*PFD complex using *in silico* approach. The top threading templates used by I-TASSER to generate individual structural models of *Pf*PFD subunits 1 to 6 are as follows: PFD from *Pyrococcus horikoshii* OT3, chain C (PDB ID: 2ZDI; for *Pf*PFD1, *Pf*PFD3, and *Pf*PFD5); and, PFD-beta subunit from *Thermococcus* strain KS-1, chain A (PDB ID: 2ZQM; for *Pf*PFD2, *Pf*PFD4, and *Pf*PFD6) (9, 10), and set to submit to generate *Pf*PFD_hexamer_ complex structure by using X-Ray diffraction-based structural model of human TRiC-PFD complex as a suitable template (11). After optimal rigid-body superimposition of the generated structural model of *Pf*PFD_hexamer_ with *Hs*PFD, the overall Root-Mean-Square Deviation value of the C-alpha atomic co-ordinates was found to be 1.45 Å, suggesting a reliable 3D structure. Assessment of the stereochemical quality and accuracy of the generated structural model of *Pf*PFD_hexamer_ displayed 91.8% of amino acid residues lying in the most favored (core) regions, with 6.8%, 0.9%, and 0.5% residues in additional allowed, generously allowed, and disallowed regions of Ramachandran plot, respectively.

*Pf*PFD_hexamer_ structural model revealed a strong resemblance with its counterparts from other eukaryotes, forming a hetero-hexamer with a jellyfish-like architecture composed of two canonical classes of subunits: α (PFD subunits: 3 and 5) and β (subunits: 1, 2, 4, and 6), arranged in the following manner -PFD3-PFD2-PFD1-PFD5-PFD6-PFD4- (11, 12) (Fig. 2*D*). The core body of *Pf*PFD_hexamer_ was found to consist of a double beta-barrel assembly, with six long tentacle-like coiled coils protruding from it in a regularly structured fashion. The comparable RMSD value and Ramachandran plot characteristics confirmed the reliability of the *Pf*PFD_hexamer_ complex to be taken further for *in silico* and *in vitro* interaction analysis and inhibition studies.

### Identification of BPD as a potential binder of *Pf*PFD_hexamer_

**Figure 4:**
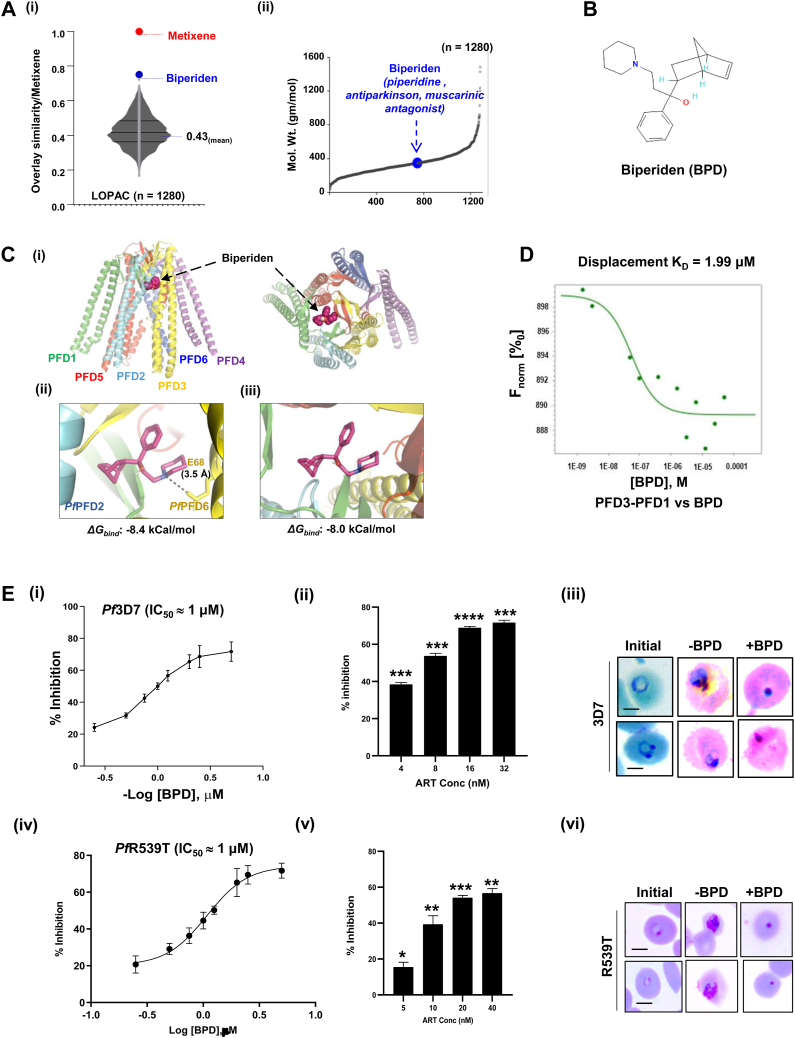
**Identification of a drug ‘Biperiden’ targeting prefoldins and its effect on artemisinin-sensitive (*Pf*3D7) and -resistant parasite (*Pf*Kelch**^**R539T)**^**.***A* (i, ii), *in silico therapeutic repositioning approach.* LOPAC^1280^ library was screened based on structural similarities with the Protein Folding Activity of Ribosomes (PFAR) inhibitor, Metixene. BPD was identified with an overlay structural similarity index of 0.75. *B*, schematic diagram of the BPD molecule. *C*, *possible architecture of the PfPFD-BPD*. The complex was generated using the modeled structure of the *Pf*PFD complex. BPD was found to interact with *Pf*PFD complex *via* two different conformations. (i) In conformation 1, BPD engaged at the interface of *Pf*PFD subunits 2 and 6, with free binding energy (*ΔG*_*bind*_) of −8.4 kCal/mol. (ii) In conformation 2, BPD was found to interact with the double beta-barrel assembly of the *Pf*PFD complex, with *ΔG*_*bind*_ of −8.0 kCal/mol. *D*, *BPD competes with PfPFD1 for binding to PfPFD3.* Competing the *Pf*PFD3-*Pf*PFD1 interaction with BPD resulted in a decrease in the MST signal starting at 893 to 888.1 units (for *Pf*PFD3-*Pf*PFD1), thus enhancing the K_D_ values to 1.99 μM. *E*, *in vitro* anti-plasmodial activity of BPD in artemisinin sensitive (*Pf*3D7) (i) and resistant parasite (*Pf*Kelch^R539T^) (iv). Ring stage *Pf*3D7 was treated with varying concentrations (250 nM to 5 μM) of BPD for 72 h. BPD inhibited the growth of the malaria parasite and displayed a potent anti-plasmodial effect with an IC_50_ value of ∼1 μM in artemisinin sensitive and resistant parasite. Error bars in line graph define measurements (mean ± SD values) calculated for each data point from three independent experiments performed in triplicates each time. (ii, v) Bar graph plots depicting the percentage growth inhibition of parasite after treatment with different concentrations of artesunate in *Pf*3D7 (ii) and resistant *Pf*Kelch^R539T^ (v). Error bars in bar graph represent measurements (mean ± SD values) calculated for each concentration of artesunate treatment in three independent experiments performed in triplicates. The *p* values were calculated by Student’s *t* test (*p*-values ≤ 0.05 ∗, *p*-value ≤ 0.01∗∗, *p*-value ≤ 0.001∗∗∗, *p* < 0.0001∗∗∗∗). (iii, vi) Light microscopy-based Giemsa-stained images of artemisinin sensitive (*Pf*3D7, iii) and resistant parasites (*Pf*Kelch^R539T^, vi) pre- and post-72 h, showing the formation of pyknotic bodies in BPD treated parasite (Scale bar = 2 μm).

The *in silico* therapeutic repositioning approach which we adopted to screen the LOPAC^1280^ library based on similarities with one of the potent Protein folding activity of the ribosome (PFAR) inhibitors, Metixene, generated BPD, with an overlay structural similarity index of 0.75 (Fig. 4*A* i). Similar to Metixene, BPD (IUPAC name: 1-{bicyclo[2.2.1]hept-5-en-2-yl}-1-phenyl-3-(piperidin-1-yl)propan-1-ol) is a member of piperidines and has a role as an antiparkinson drug and a muscarinic antagonist (Fig. 4*A* ii). In a recent investigation, BPD along with 16 other FDA-approved drugs, were identified to harbor anti-prion activity (13). In the study, seven out of the 17 compounds with lower IC_50_ values were further examined for their ability to inhibit PFAR. However, because of its high IC_50_ value, the PFAR activity of BPD was not investigated further. Given the fact that the structural features of BPD are similar to those of the PFAR-active compound, Metixene, we hypothesized that BPD would interact with additional genes whose activity is associated with protein folding and show an inhibitory effect, which prompted us to investigate the direct interaction of BPD with *Pf*PFD.

A plausible architecture of the *Pf*PFD-BPD complex was constructed using the generated structural model of the *Pf*PFD_hexamer_. Structural representation of BPD is depicted in Figure 4*B* and a schematic representation of complex formation between *Pf*PFD_hexamer_ and BPD is shown in Figure 4*C* i. BPD was found to interact with *Pf*PFD_hexamer_ *via* two different conformations. In conformation 1, BPD was found to engage at the interface of *Pf*PFD subunits 2 and 6, with free binding energy (*ΔG*_*bind*_) of −8.4 kCal/mol, *via* polar contact (H-bonds) with Glu^68^ *Pf*PFD6 with a bond length of 3.5 Å (Fig. 4*C* ii). Contrastingly, in conformation 2, BPD was found to interact with the core body of the *Pf*PFD complex consisting of a double beta-barrel assembly, although with a lower *ΔG*_*bind*_ of −8.0 kCal/mol (Fig. 4*C* iii). It was, therefore, hypothesized that *Pf*PFD complexation with BPD in either of the conformations would result in a diminished ability to bind and stabilize newly synthesized proteins, thereby impairing the correct folding of the nascent polypeptides.

To further evaluate whether BPD can inhibit the interaction of prefoldin subunits, we performed an MST-based competition experiment, in which *Pf*PFD3 was used as a labeled protein; and, BPD and *Pf*PFD1 as the unlabelled competing ligands. The interaction of *Pf*PFD3 with *Pf*PFD1 exhibited increasing MST signals (F_norm_ [%ₒ]) starting at 886.1 to 892.1 units (for *Pf*PFD1), resulting in the K_D_ value of 81.6 nM (Fig. 2*C* i). Competing the interactions with BPD resulted in a decrease in the MST signal starting at 893 to 888.1 units, thus enhancing the K_D_ value to 1.99 μM (Fig. 4*D*). The difference in the MST signal in the absence and presence of BPD indicated that it competes with *Pf*PFD1 for binding to *Pf*PFD3.

### BPD treatment inhibits *in vitro* growth of *P. falciparum*

BPD was evaluated for its growth-inhibitory effect on *Pf*3D7 and *Pf*3D7k13^R539T^ using an *in vitro* intra-erythrocytic Growth Inhibition Assay (GIA), wherein, parasitemia levels were determined at 72 h post-treatment. BPD inhibited the growth of the malaria parasite and displayed a potent anti-plasmodial effect with an IC_50_ value of ∼1 μM in *Pf*3D7 and *Pf*3D7k13^R539T^ (Fig. 4*E* i, iv). Artesunate served as a control, which is a well-known anti-malarial drug, and killed ∼50% of the parasites at a concentration of 8 nM and 20 nM in *Pf*3D7 and *Pf*3D7k13^R539T^ respectively. (Fig. 4*E* ii, v). Giemsa-stained images of BPD-treated and untreated parasites are shown in Figure 4*F* iii, vi.

### *PfP*FD2 interacts with *Pf*α-tubulin-I and their interaction is inhibited by BPD

**Figure 5:**
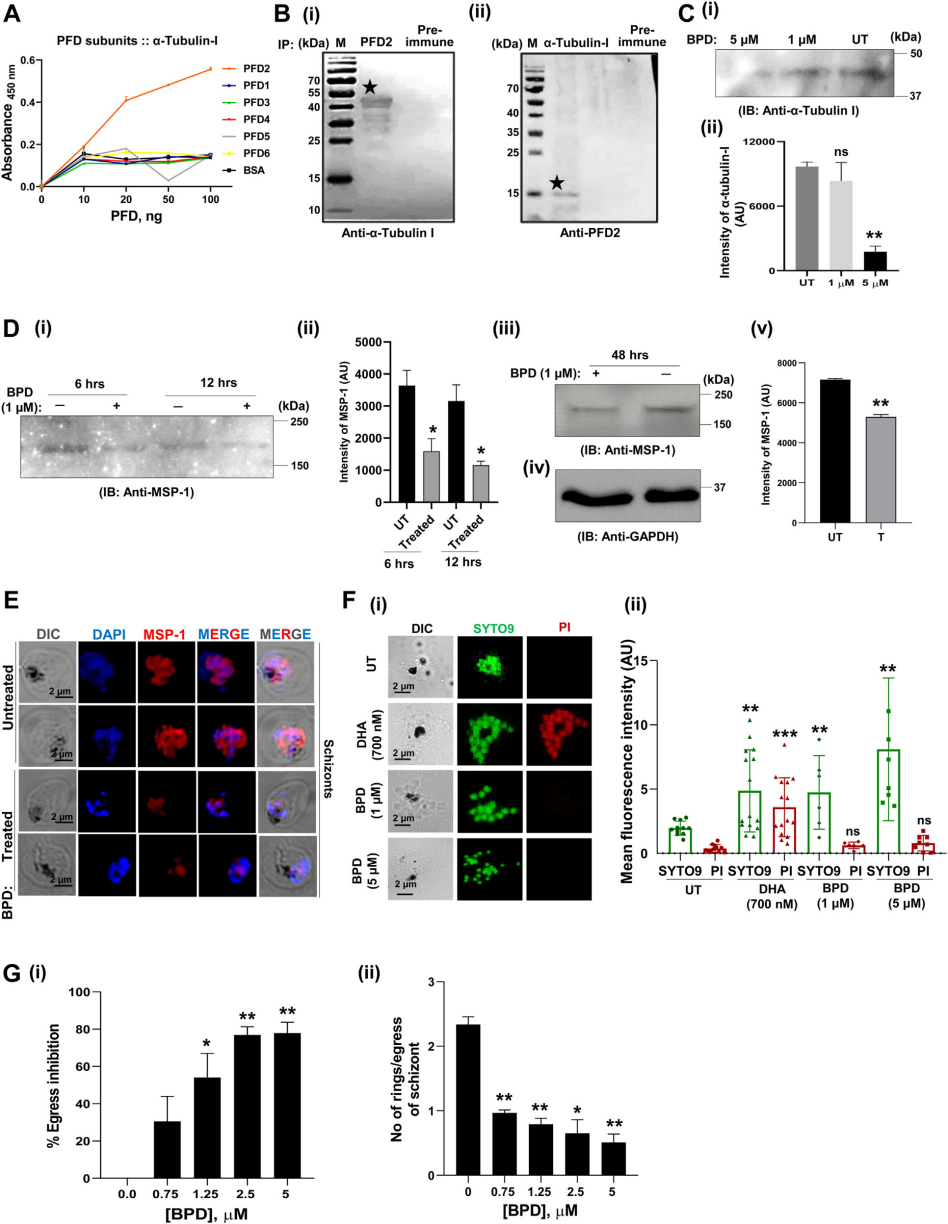
**Interaction studies of *PfP*FD2 with *Pf*α-tubulin-I and effect of BPD on native *Pf*α-tubulin-I and *Pf*MSP1 expression.***A*, *PfPFD2 interacts with Pfα-tubulin-I.* Semi-quantitative ELISA was done in which *Pf*PFD2 upon titration with *Pf*α-tubulin-I depicted the interaction in a concentration-dependent manner. The experiments were performed in three independent biological replicates done in triplicate each time, and in line graph shown with error bars define mean ± SD values calculated for each data point. *B*, *Co-IP-based interaction analysis of PfPFD2 and Pfα-tubulin-I.* (i) *Pf*PFD2 antiserum was cross-linked to the AminoLink plus Coupling Resin, followed by incubation with the parasite lysate prepared from the mix-stage parasite population. The desired protein band of *Pf*α-tubulin-I was observed in the eluted fraction. (ii) Similarly, reverse co-IP confirmed the interaction between the two proteins. *C*, *BPD destabilizes Pf*α-tubulin-I. (i) Western blot analysis demonstrated that the *Pf*α-tubulin-I levels get markedly reduced upon the treatment of the parasites with BPD for 6 h (1 μM and 5 μM). (ii) Bar graphs representing the intensity plots of *Pf*α-tubulin-I in treated and untreated samples. AU depicts arbitory units. (*p*-value ≤ 0.01∗∗; ns-non significant). *D*, *BPD destabilizes PfMSP-1.* (i) Western blot analysis demonstrating reduction in *Pf*MSP-1 expression level in BPD-treated parasites at different time points of BPD treatment (1 μM; 6 h, 12 h). (ii) Intensity of *Pf*MSP1 under each condition is shown as an intensity graph (*p*-values ≤ 0.05 ∗). AU depicts arbitory units. (iii) Western blot analysis demonstrating reduction in *Pf*MSP-1 expression level in BPD-treated parasites at 48 h of BPD treatment. (iv) Western blot showing GAPDH as loading control in the assay. (v) Intensity plot of *Pf*MSP-1 levels in the parasite treated with BPD for 48 h. (*p*-value ≤ 0.01∗∗). AU depicts arbitory units. Data represented in *C* (i) and *D* (i, iii) were obtained from two independent biological replicates and a representative blot is shown in each case. Error bars in intensity plots (*C* ii, *D* ii, v) define measurements (mean ± SD) taken from two independent experiments. *E*, confocal microscopy-based analysis demonstrated reduced expression of *Pf*MSP-1 in the BPD-treated schizonts (Scale bar =2 μm). *F*, *effect of BPD on parasite viability*. Trophozoite-parasitized RBCs were treated with BPD (1 and 5 μM) and DHA (700 nM) for 6 h and post-treatment, parasites were subjected to PI/SYTO9 (*Red/Green*) co-staining. (i) Microscopic imaging data revealed that the BPD-treated parasites were SYTO9-positive and PI-negative, suggesting that the viability of the parasites was not compromised upon BPD-treatment. DHA treated parasites served as positive control while untreated parasites were taken as negative control. Viability experiments using SYTO9/PI were performed in three independent biological replicates and representative images of a biological repeat are shown. (Scale bar =2 μm). (ii) Mean fluorescence intensity of SYTO9 and PI in BPD, DHA treated and untreated parasites (n = 10) are plotted and shown as bar graphs. AU depicts arbitory units. Error bars define standard deviation among no. of cells taken for intensity measurements (n = 10). The *p* values were calculated by Student’s *t* test (*p*-value ≤ 0.01∗∗, *p*-value ≤ 0.001∗∗∗, ns-non-significant). *G*, *BPD inhibits egress and invasion of the parasite.* (i) Bar graph plot representing percent egress inhibition at different concentrations of BPD treatment. BPD significantly inhibited the egress of the parasite by ∼60% at 1.25 μM, and ∼75% at 2.5 μM and 5 μM concentrations. The *p* values were analysed by Student’s *t* test (*p*-values ≤ 0.05 ∗, *p*-value ≤ 0.01∗∗). (ii) Bar graph plot showing no. of rings per egress of schizont at different concentrations of BPD treatment. The number of rings formed per egress of schizont was also significantly reduced in the presence of BPD, as compared to the control. The *p* values were calculated by Student’s *t* test (*p*-values ≤ 0.05 ∗, *p*-value ≤ 0.01∗∗). Egress and invasion experiments were performed in three independent biological replicates and error bars in bar graph plots depict measurements (mean ± SD) calculated from three independent replicates.

Previous reports in eukaryotes indicate that PFD interacts with cytoskeletal proteins (14, 15, 16). Protein-Protein Interaction data available in the PlasmoDB database also indicate the interaction of *Pf*PFD2 with *Pf*α-tubulin-I. To validate the interaction, preliminary screening was carried out with semi-quantitative ELISA, wherein, *Pf*PFD2 upon titration with *Pf*α-tubulin-I depicted the interaction in a concentration-dependent manner (Fig. 5*A*). The interaction of *Pf*PFD2 with *Pf*α-tubulin-I was confirmed with co-IP, in which *Pf*PFD2 antiserum was cross-linked to the AminoLink plus Coupling Resin, followed by incubation with the parasite lysate prepared from the mix-stage parasite population. The desired protein band of *Pf*α-tubulin-I was observed in the eluted fraction (Fig. 5*B* i). Similarly, reverse co-IP, in which *Pf*α-tubulin-I antisera was cross-linked to the resin, followed by incubation with the parasite lysate, confirmed the interaction between the two proteins (Fig. 5*B* ii). Collectively, these findings point to a possible interaction between *Pf*PFD2 and *Pf*α-tubulin-I. Further, Western blot analysis demonstrated that the *Pf*α-tubulin-I levels get markedly reduced upon the treatment of the parasites with BPD (Fig. 5*C* i), suggesting the inhibitory effect of BPD in blocking *Pf*PFD2 interaction with its substrate protein *Pf*α-tubulin-I. The full uncropped blot is represented in Fig. S2. Band intensity of *Pf*α-tubulin-I before and after BPD treatment is given in Figure 5*C* ii.

### BPD destabilizes *Pf*MSP-1, a substrate of *Pf*PFD

We previously reported that Merozoite surface protein-1 (*Pf*MSP-1), a critical protein involved in the egress and invasion of the parasite, is a substrate of *Pf*PFD6 (17). To evaluate how BPD affects the function of *Pf*PFD subunits, the expression, and localization of *Pf*MSP-1 were assessed in the BPD-treated parasites. Expression of *Pf*MSP-1 was tested at two different time points (6 h and 12 h) of drug treatment (1 μM) using western blotting. Our results indicate reduced *Pf*MSP-1 expression with prolonged drug treatment (Fig. 5*D* i; Full uncropped blots are represented in Fig. S2). Band intensity of *Pf*MSP-1 obtained in untreated and BPD treated parasite are plotted in Figure 5*D* ii. Furthermore, *Pf*MSP-1 expression gets reduced when parasites were treated with BPD (1 μM) for 48 h, (Fig. 5*D* iii), which is also shown by plots of band intensity of treated and untreated samples (Fig. 5*D* v). GAPDH served as a loading control (Fig. 5*D* iv). Confocal microscopy validated the western-based analysis, wherein, reduced expression of *Pf*MSP-1 was observed in the BPD-treated schizonts (Fig. 5*E*); NapL served as a control (Fig. S5). These findings imply that BPD destabilizes *Pf*MSP-1 expression by interacting with *Pf*PFD.

To further confirm the specific effect of BPD on MSP-1 expression, the viability of the BPD-treated parasites was further assessed by co-staining the parasites with Propidium Iodide (PI) and SYTO9. PI is a nuclear counterstain that fluoresces red and is used to identify dead cells in a cell population; and, SYTO9 is a green fluorescent nucleic acid that stains both live and dead cells. Our data suggest that BPD exposure for 6 h didn’t have any cytotoxic effect (Fig. 5*F* i and ii), and further suggest the specific inhibitory potency of BPD on MSP1 expression.

We next evaluated the effect of BPD on the egress and invasion of the parasite. BPD significantly inhibited the egress of the parasite by ∼60% at 1.25 μM, and ∼75% at 2.5 μM and 5 μM concentrations (Fig. 5*G* i). Similarly, the number of rings formed per egress of schizont was significantly reduced in the presence of BPD, as compared to the control (Fig. 5*G* ii). This indicates that disturbing the *Pf*PFD-mediated proteostasis by BPD inhibits the egress and invasion processes of the parasite.

### Complementation of *Pf*PFD-6 in yeast mutants restores growth and reverts to artemisinin resistance

**Figure 6:**
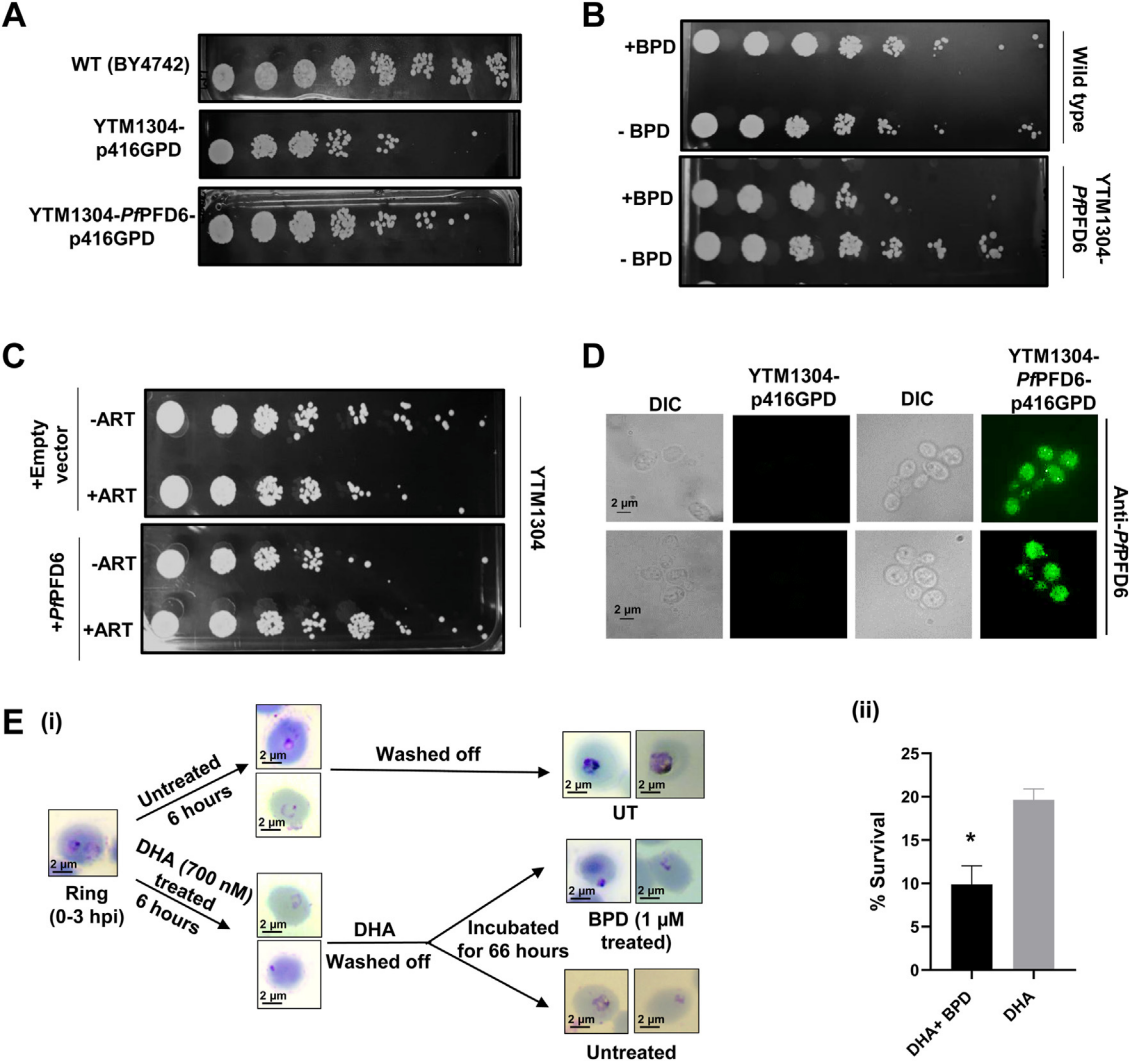
**Complementation of *Pf* Prefoldin-6 in yeast orthologous system and effect of BPD and artemisinin in mutant yeast strains.***A*, complementation with *Pf*pfd6 rescues YTM 1304 cell growth as depicted using Spot assays. Cultures of wild-type (BY4742), *Sc*pfd6Δ (YTM 1304), and *Sc*pfd6Δ complemented with *Pf*PFD6 were harvested and adjusted to an optical density, A600 of 0.1 using sterile YPD media. Serial 10-fold dilutions of each culture were spotted onto YPD agar plates. Upon complementing YTM 1304 with *Pf*pfd6, partial restoration of cell growth was observed. *B*, *Pf*PFD6-targeting effect of BPD. The growth pattern of YTM1304 and YTM1304 complemented with *Pf*PFD6 was analyzed in the presence and absence of BPD. Cultures were harvested and adjusted to A600 of 0.1 using sterile YPD media. Serial 10-times dilutions of each culture were spotted onto solid YPD-agar plates and incubated for 2 days at 30 °C. Interestingly, *Pf*PFD6-complemented yeast exhibited reduced growth in the presence of BPD as compared to the control (untreated). *C*, effect of artemisinin on the growth pattern of YTM1304 complemented with *Pf*PFD6. The yeast cells YTM1304-p416GPD, and YTM1304-p416GPD-*Pf*PFD6 were grown overnight and cultures were diluted to initial A_600_ = 0.1. Cultures were allowed to grow for 12 h in the presence and absence of artemisinin (8 μM). Each culture was harvested and adjusted to an A600 value of 0.1. Subsequently, it was 10-fold serially diluted and was spotted onto a YPD agar plate followed by incubation at 30 °C for 2 days. *Pf*PFD6 renders the mutant cells less sensitive to artemisinin. *D*, immunofluorescence images showing the expression of *Pf*PFD6 in mutant yeast strains (YTM1304) transformed with *Pf*PFD6-p416GPD. Expression was not observed in mutants transformed with only p416GPD. (Scale bar = 2 μm). *E*, (i) Schematic representation showing the methodology followed for ring survival assay, wherein ART-resistant ring staged *Pf*3D7k13^R539T^ parasites were treated with DHA followed by treatment with BPD. The surviving parasites were scored and the percentage survival was calculated for each condition. (Scale bar = 2 μm). (ii) Bar graph plot depicting the percent survival of DHA and BPD treated rings and its comparison with DHA alone treated *Pf*3D7k13^R539T^ parasites. BPD potently decreases the survival of DHA pretreated resistant rings when compared to DHA alone. Ring survival assays were carried out in two independent biological replicates. Values in error bars represent mean ± standard deviation among two independent experiments. (*p*-values ≤ 0.05 ∗).

To ascertain the identity and function of *P. falciparum* Prefoldins, we exploited *S. cerevisiae* orthologous model. Functional characterization was done in a yeast orthologous model by expressing *Pf*PFD6 in cells harboring mutants. We cloned *Pf*PFD6 in p416-GPD expression vector, followed by transformation of the resulting plasmid (p416-GPD*-Pf*PFD6) in *Scpfd6*-deleted strain of *S. cerevisiae* (YTM1304). Following transformation, spot assay analysis was performed that revealed restoration of cell growth in YTM1304 complemented with *Pf*PFD6 (YTM1304-*Pf*pfd6), as depicted in (Fig. 6*A*). WT (BY4742) was used as a positive control in the assay.

Having demonstrated the restored growth of YTM 1304 upon complementation with *Pf*PFD*6*, we next investigated BPD specificity for *Pf*PFD6. Two sets of yeast cultures (Wild type and YTM 1304-*Pf*PFD6) were used in the assay and were treated with 20 μM BPD to assess their growth patterns through spot assay analysis. Interestingly, YTM 1304-*Pf*PFD6 exhibited reduced growth in the presence of BPD as compared to the control (untreated) (Fig. 6*B*). However, no significant difference was observed in BPD-treated and untreated wild-type *S. cerevisiae* (Fig. 6*B*). These results suggest that the decline in growth is primarily attributed to the targeted effect of BPD on *Pf*PFDs. Further, to elucidate the effect of artemisinin, two batches of yeast cultures were cultivated: one comprising YTM 1304-p416-GPD and the other consisting of YTM 1304-*Pf*PFD6. These cultures were grown in the presence and absence of ART. Our Spot assay analysis showed the reduced growth of YTM1304-p416-GPD in the presence of ART, while, complemented strain YTM 1304-*Pf*PFD6 exhibited enhanced growth (Fig. 6*C*). These data point out that *Pf*PFD6 has a significant role in the development of resistance against artemisinin.

We also checked the expression of *Pf*PFD6 in YTM 1304 mutants transformed with *Pf*PFD6-p416GPD through immunofluorescence assays. Our data suggests the expression of *Pf*PFD6 in transformed yeast cells (Fig. 6*D*). We could not observe any expression in YTM 1304 mutants transformed with empty vector p416GPD, suggesting the specificity of our functional complementation assays.

### BPD inhibits the growth of ART-resistant parasites recovered after DHA treatment

Since our previous results suggest the gain of artemisinin resistance on complementing *Pf* prefoldin in yeast, we tested whether BPD has any inhibitory potential on ART-resistant parasites recovered after DHA treatment. *In vitro* studies involve the use of ring-stage survival assay (RSA) that are considered golden standard for *in vitro* measurement of artemisinin resistance. In RSA, the parasites are tightly synchronized in order to assay during the short window (0–3 h rings) that can differentiate ART-resistant parasites from ART-sensitive ones (Fig. 6*E* i). We employed this assay in ART-resistant line *Pf*3D7k13^R539T^ to investigate the effect of BPD on artemisinin survivors. The percentage survival of DHA pretreated rings decreased in the presence of BPD (Fig. 6*E* ii), suggesting that BPD is potent in eliminating resistant parasites surviving post-DHA treatment. These data further shed light on the role of prefoldins in artemisinin-resistant mechanism.

### BPD inhibits parasite growth *in vivo*

**Figure 7:**
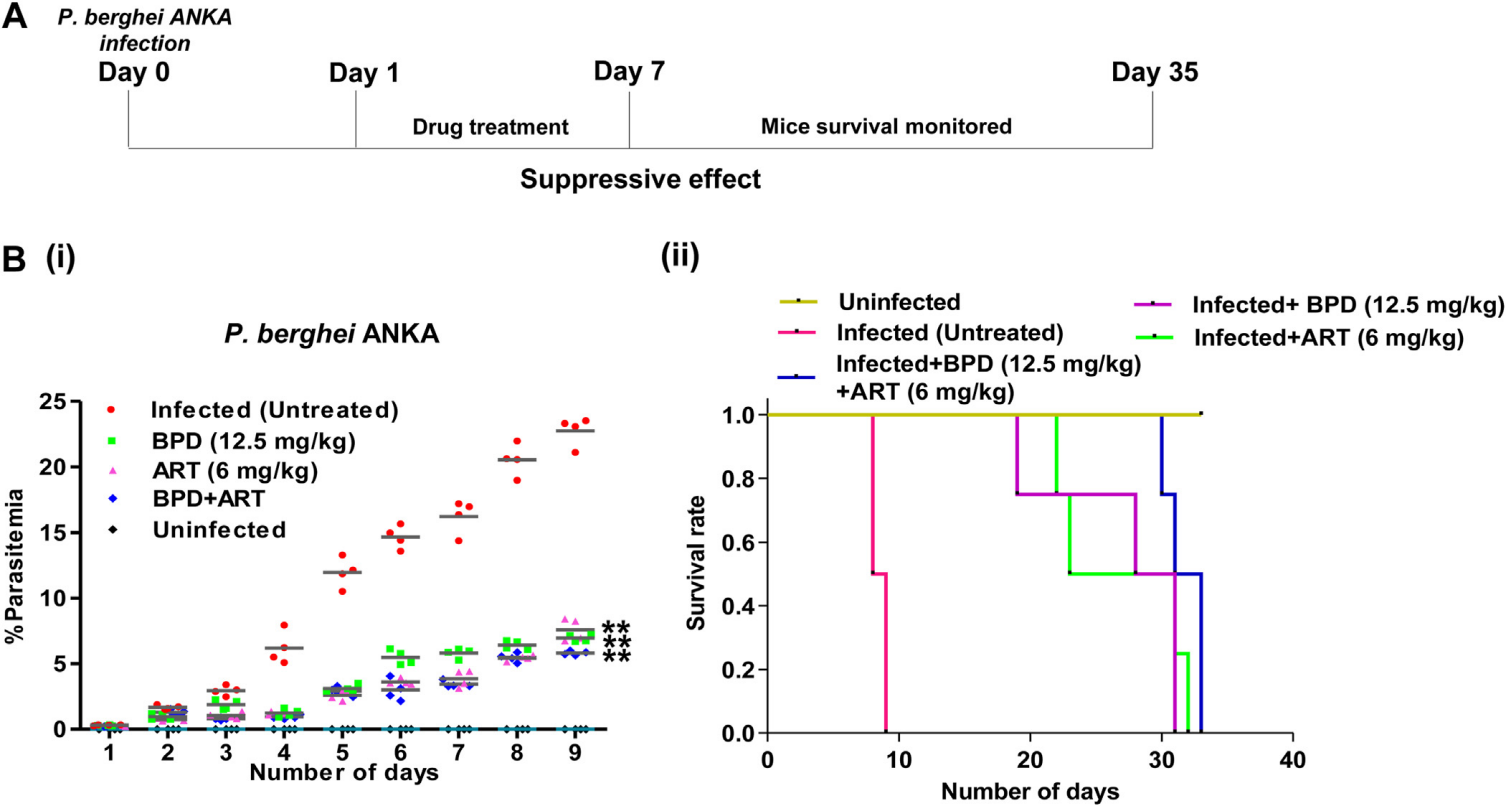
**BPD inhibits the growth of the rodent malaria parasite and increases the survival rate.***A*, *schematic representation of the methodology used for dose administration of BPD in mice*. *B*, *in vivo anti-malarial activity of BPD.* (i) Graph showing percent parasitemia in five experimental groups. On day 9, parasite load in the untreated group was ∼23%, whereas, in BPD- and artesunate-treated group, ∼7% parasitemia was observed. (ii) Graph showing percent survival of *P. berghei*-infected mice. On day 9, all mice of the untreated group died while more than 50% of mice survived for 20 days in the treated group. Statistical significance was calculated for treated groups and compared with untreated group of mice, and is shown with *p*-value ≤ 0.01∗∗.

The anti-plasmodial activity of BPD was also evaluated *in vivo*, in mice infected with *P. berghei* ANKA. A schematic representation of the methodology followed for the experiment is shown in Figure 7*A*. On the ninth day post-infection, parasitemia in the untreated mice reached over 23%, and all mice died (Fig. 7*B* i). However, the parasitemia was around 7% in infected mice treated with BPD or artesunate (Fig. 7*B*i). On the 23rd day post-infection, survival of the infected mice treated with BPD was found to be 50% (Fig. 7*B* (ii)). These findings suggest that BPD-treated mice possess reduced parasitemia and a higher survival rate than the untreated ones.

## Discussion

Artemisinin resistance has become a major threat to the efficacy of artemisinin-based combination therapies. Although artemisinin resistance has become prevalent, there is less knowledge about the molecular mechanisms that make *P. falciparum* insensitive to artemisinins. A study by Mok *et al.* carried out transcriptome analyses of 1043 clinical *P. falciparum* isolates and identified the underlined mechanisms on the transcriptional level that mediate artemisinin resistance (5). The same study reported that artemisinin resistance is linked with the coordinated transcription of several chaperone partners including the unexplored Prefoldins of *P. falciparum*. In light of these facts, the present study attempted to elucidate the functions of *Pf* Prefoldins in malaria parasites and their relevance to artemisinin resistance. This piece of knowledge can fill the key gaps to malaria biology and can help to combat artemisinin resistance.

Since *Pf*K13 protein is reported to be responsible for mediating ART resistance, we performed Co-IP studies coupled with mass spectrometry analysis to identify the possibility of interaction between *Pf*K13 and *Pf* Prefoldin. The protein-protein interaction network generated using STRING suggests the interaction of *Pf*K13 with Prefoldins. This interaction was further confirmed by SPR and co-IP studies. These data prompted us to explore the role of Prefoldin in *Pf* and their contribution towards artemisinin resistance.

Abnormalities in protein synthesis, folding, or clearance disrupt cellular processes, leading to pathological consequences. To maintain a functional proteome, cells rely on a complex network of surveillance mechanisms directed by molecular chaperones, which fold newly synthesized polypeptides, refold misfolded proteins, and guide protein degradation (18). Genomic cataloging reveals that approximately 2% of the *P. falciparum* genes encode for an extensive array of molecular chaperones that are believed to play a crucial role in assisting the parasite to adapt to infection-induced stress (19). Investigating chaperones in the parasitic system, therefore, may not only provide potential therapeutic targets but also offer insights into novel principles of chaperone function in parasite biology. Among the chaperones, Prefoldin, a highly acclaimed co-chaperone protein, aids in the proper folding of essential proteins. Prefoldin has garnered considerable attention for its significance in emerging domains such as nanoparticles, biomaterials, and tumor biology. Furthermore, each subunit of Prefoldin possesses distinct and independent roles apart from its involvement in the Prefoldin complex (8). Although archaea and eukaryotes have been well-characterized in terms of Prefoldin function, *Plasmodium* species lack sufficient studies investigating the potential importance of Prefoldin subunits in managing the proteostasis of critical plasmodial proteins. The transcriptome data obtained from PlasmoDB indicates the presence of all six Prefoldin subunits in *Plasmodium* (PF3D7_1107500, PF3D7_1416900, PF3D7_0718500, PF3D7_0904500, PF3D7_1128100, and PF3D7_0512000). CDS for the full-length Prefoldin subunits were cloned into a bacterial expression system and subsequently overexpressed and purified. Mice were immunized to generate polyclonal antibodies against each Prefoldin subunit. These antibodies exhibited specific binding to the corresponding subunits, enabling their detection and analysis. qRT-PCR and Western blot analysis confirmed the expression of Prefoldin subunits at both transcript and protein levels during the intra-erythrocytic stages of the malaria parasite. Also, expression of Prefoldin subunits was observed to be upregulated in artemisinin-resistant strains as compared to sensitive parasites, underlining their importance during resistant mechanism. Furthermore, our immunofluorescence-based experiments illustrate the localization of Prefoldin subunits within the cytosol of the parasite. Notably, the co-localization of Prefoldin subunits with the *Plasmodium* cytosolic marker protein '*Pf*NapL' reveals a substantial overlap of localization signals, indicating their coexistence within the parasite cytosol. These findings align with previous reports in archaea and eukaryotes, suggesting the presence of the Prefoldin complex in the cytoplasm (20). Moreover, this suggests that the role and localization of Prefoldin subunits may be conserved across different organisms.

The Prefoldin complex, which functions as a cytoplasmic chaperone protein, is assembled through the interaction of its six distinct subunits, resulting in the formation of a hybrid oligomer. Based upon previous research (8), we conducted Nano temper-based interaction analysis, revealing the high affinity of PFD3 for PFD1 and PFD2, while PFD5 exhibited greater affinity towards PFD2, PFD4, and PFD6. These data suggest the ability of *Pf*PFDs to interact with each other and form a complex.

To gain insights into the role of the *Pf*PFD complex and explore potential antimalarial small molecules, we conducted an analysis of various chemotypes approved by the FDA and identified biperiden as a probable *Pf*PFD-binding molecule. *In silico* analysis suggested that BPD binds to the PFD complex, demonstrating a significant one-to-one interaction of *Pf*PFD subunits with BPD. Our competition experiments using MST revealed disruption of protein-protein interactions involving the Prefoldin subunits by BPD. Further, through parasite growth inhibition assay, we evaluated the effect of BPD treatment on the *in-vitro* growth of *P. falciparum in* artimisinin-sensitive *Pf*3D7 and resistant line *Pf*3D7k13^R539T^, and observed an IC_50_ of 1 μM, indicating its efficacy in inhibiting parasite growth in both strains.

Actin and tubulins are highly abundant cytoskeletal proteins that play crucial roles in numerous cellular functions, including cellular mobility, morphogenesis, polarity establishment, cell division, and intracellular transport. Considering the importance of these functions, we observed that *Plasmodium* Prefoldin also interacts with α-tubulin-I, thus supporting the crucial role of Prefoldin abundance in microtubule function. This is consistent with the previous findings in *Arabidopsis*, wherein, *Pfd1-6* mutant showed considerable microtubule defects, including oryzalin hypersensitivity, impaired cell division, cortical array disorganization, and reduced microtubule dynamics (21).

Previously, we reported the interaction of *Pf*PFD6 with MSP-1 (17). Moreover, treatment with BPD efficiently impeded the interaction between the PFD complex and its substrate, resulting in the degradation of α-tubulin-I and MSP-1. These proteins are known to be essential for the growth and proliferation of the parasite, prompting us to investigate the impact of Prefoldin inhibition on parasite growth and proliferation. Our results demonstrated that treatment with BPD led to the inhibition of parasite egress and reduced the formation of rings per schizont egress. Understanding the processes involved in *Plasmodium* egress inhibition could pave the way for the development of new pharmacological targets and more effective antimalarial therapies.

We next used a yeast orthologous system to show that *Pf*PFD-6 complementation in yeast mutants restores the growth of the mutant strain. The yeast model system was also used to show the selectivity of the compound BPD for *Pf* Prefoldins. Interestingly, we observed that Prefoldin decreases the sensitivity of artemisinin in yeast, providing evidence for the role of PFDs in providing resistance to *Pf*.

Clinical ART resistance is defined as a parasite clearance half-life ≥ 5 h from a patient’s blood post-treatment. To measure ART resistance *in vivo*, assays that measure the linear decline of parasitemia in patients after drug treatment are used (22). *In vitro* studies involve the use of ring-stage survival assay (RSA) that is considered the golden standard for *in vitro* measurement of artemisinin resistance. Here, the parasites are tightly synchronized in order to assay during the short window (0–3 h rings) that can differentiate ART-resistant parasites from ART-sensitive ones. We employed this assay in ART-resistant line *Pf*3D7k13^R539T^ to investigate the effect of BPD on artemisinin survivors. Our data clearly demonstrated that artemisinin-resistant parasites can be inhibited by treatment with Prefoldin targeting molecule BPD. These observations further shed light on the unexplored role of *Pf* Prefoldins in the artemisinin resistance mechanism.

Assessing the *in vivo* efficacy of a drug is crucial for identifying promising leads in drug development. Therefore, we also evaluated the *in vivo* efficacy of BPD in a rodent malaria model. The results showed that the drug efficiently inhibited parasite development and increased the survival rate of mice at a dosage of 12.5 mg/kg.

**Figure 8:**
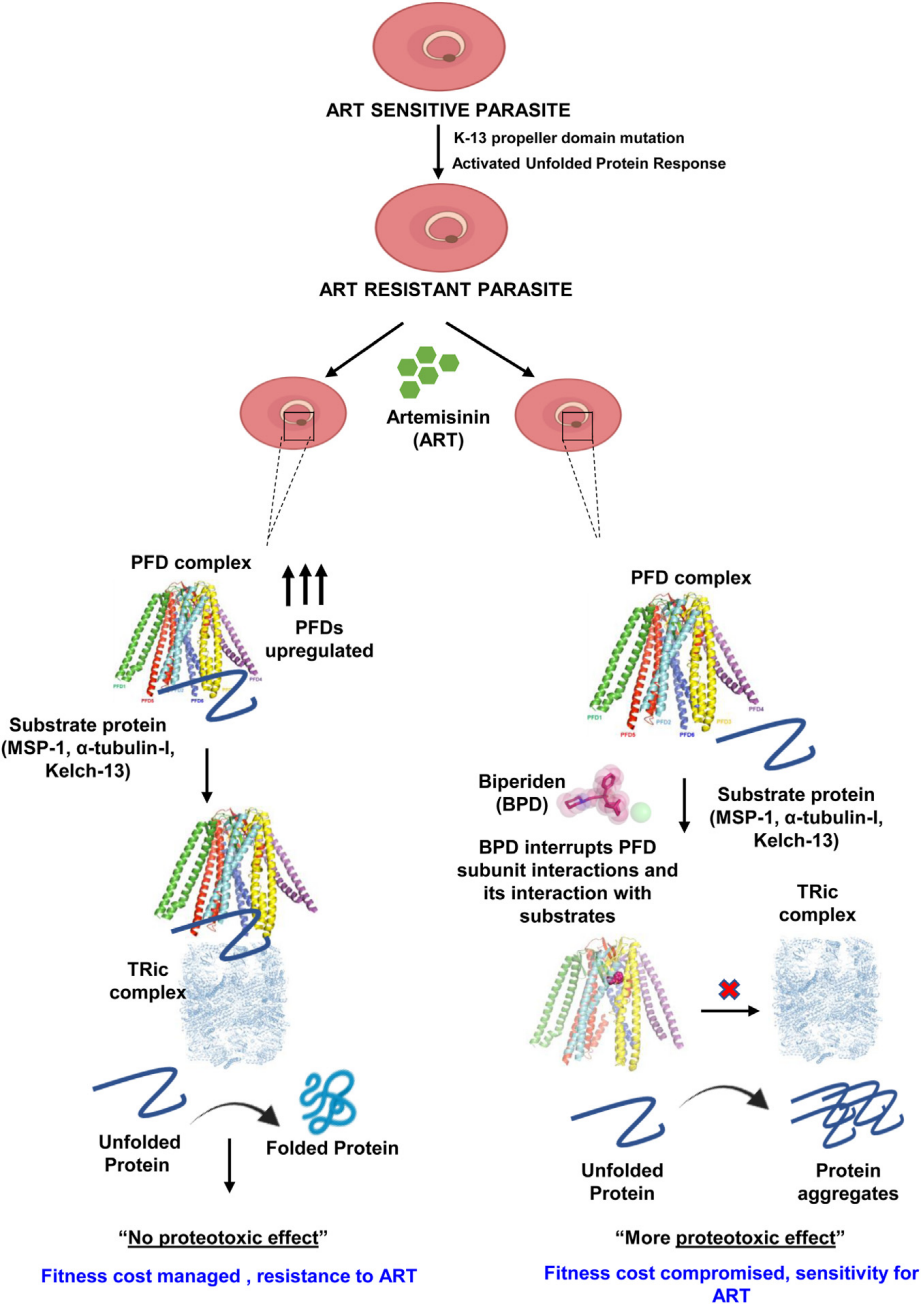
**Model depicting the role of Prefoldin in providing artemisinin resistance to parasites, and probable action of BPD targeting this process to overcome resistance.** Prefoldin upregulation in resistant parasites enhances their interaction with α-tubulin-I and *Pf*MSP1, and is responsible for the transfer of nascent α-tubulin-I polypeptide and *Pf*MSP1 to the TRiC/CCT complex, ensuring the accurate folding of proteins. As a consequence, there is no proteotoxic effect, and fitness cost is managed. On the contrary, targeting Prefoldins with BPD disrupts its interaction with substrate proteins and inhibits their folding process. Accumulation of protein aggregates leads to a more proteotoxic effect. Subsequently, fitness cost of resistant parasites is compromised.

Based on our results, we have proposed a model depicting the role of Prefoldins in the artemisinin resistance mechanism (Fig. 8). The model demonstrates the contribution of Prefoldin in providing artemisinin resistance to parasites, and probable action of BPD targeting this process to overcome resistance. Altogether, this study is the first to shed light on the unexplored Prefoldin subunits during the asexual stage of *P. falciparum* with special relevance to understanding their role in providing artemisinin resistance and identifying them as potential pharmacological targets. Complementation of prefoldin in yeast mutants provides evidence that *Pf*PFD6 expression in yeast leads to the gain of artemisinin resistance. Additionally, we identified the interaction between the drug molecule, BPD, and the Prefoldin subunit complex, and demonstrated its impact on the proteostasis of key interacting proteins, namely α-tubulin-I and MSP-1. Furthermore, our *in vitro* and *in vivo* results establish BPD as an anti-plasmodial inhibitor in both artemisinin-sensitive *Pf*3D7 and resistant line *Pf*3D7k13^R539T^. This research contributes to ongoing efforts in combating resistance management and reducing the burden of this deadly disease worldwide.

## Experimental procedures

### *In vitro* culture of *P. falciparum*

*P. falciparum* laboratory-adapted strain 3D7 was cultured *in vitro* using the standard protocols, as described previously (23). Briefly, the parasites were cultured in RPMI 1640 (GibcoTM) medium supplemented with 5.9 gm/L HEPES (Sigma-Aldrich), 50 mg/L hypoxanthine (Sigma-Aldrich), 2 gm/L sodium bicarbonate (Sigma-Aldrich), 5 gm/L AlbuMax I (for 3D7, R539T; GibcoTM) and 10 mg/L Gentamicin (Sigma-Aldrich). The culture was maintained in 75 cm2 culture flasks (Corning) using fresh O-positive (O+) human erythrocytes, under an ambient mixed gas environment (5% O2, 5% CO2, and 90% N2) at 37 °C. Before every experiment, the parasite culture was tightly synchronized with 5% D-sorbitol for two successive intra-erythrocytic proliferative cycles, followed by enrichment of trophozoites or schizonts parasitized erythrocytes with 65% percoll. Percoll synchronization was performed post 24 to 28 h of sorbitol treatment.

### Co-IP assays and mass spectrometry analysis

The co-IP assays were carried out using AminoLink Plus Coupling Resin (Pierce Biotechnology) following the manufacturer’s protocol. Briefly, the resin was washed with coupling buffer (0.1 M Na_3_PO_4_, 0.15 M NaCl; pH = 7.2) followed by incubation with purified anti-*Pf*K13 antibodies and preimmune sera for 2 h. Post this, the resin was washed and incubated with 50 mM sodium cyanoborohydride (NaCNBH_3_) at 4°C for overnight. The parasite lysate was then added with the resin and incubated overnight at 4 °C followed by washing with wash buffer. Bound proteins were eluted using elution buffer (0.1–0.2 M glycine-HCl; pH = 2.5–3.0) and neutralized with neutralizing buffer (1M Tris; pH = 9.0) and run on 10% SDS-PAGE. The eluted proteins were digested by using trypsin (20 μg/ml). Extracted peptides were acidified to 0.1% formic acid and assessed by Orbitrap VelosPro mass spectrometer coupled with nano-LC 1000 (Thermo Fisher Scientific Inc). Using STRING online tool, interactome of proteins were generated that showed chaperone functions.

### Expression and purification of *Pf*K13

Purification of recombinant *Pf*K13 was performed as described previously (24).

### Surface plasmon resonance (SPR)-based interaction analysis

The interaction of *Pf*K13 with *Pf*PFD6 was evaluated by the Auto LAB ESPRIT SPR instrument (Kinetic Evaluation Instruments BV). Briefly, recombinant *Pf*K13 (20 μM) was immobilized on a gold sensor chip previously activated through amine coupling. PBS was used as an immobilization and binding buffer. *Pf*PFD6 was injected in increasing concentrations (25 nM, 100 nM, 1 μM, 2.5 μM, and 5 μM) over the *Pf*PFD6-immobilized chip surface. 50 mM NaOH was used to regenerate the chip surface. Data were analyzed using Auto Lab ESPRIT kinetic evaluation software. K_D_ value was estimated using the Integrated Rate Law (IRL) equation:

$$R\left(t\right)=E.\left(1-e^{Ks.t}\right)+R\left(0\right)$$

Where E is the maximal extent of change in response at a certain concentration and is equal to k_a_.C.R_max_/(k_a_.C + k_d_); K_s_ is equal to (k_a_.C+ k_d_); and R(0) is the response unit at *t=0*. E and K_s_ were assessed at each concentration by minimizing the residual sum of squares between observed data and the model equation using Solver in MS Excel. Calculation of K_s_ at different concentrations of the ligand was performed to estimate K_D_ by using following equation:

$$K_{s}=k_{a}.C+kd$$

Where K_s_ is a concentration-dependent factor that relies on k_a_ and k_d_. The ratio of intercept (k_d_) and slope (k_a_) of the above line was determined to be the dissociation constant or K_D_.

### Cloning, expression, and purification of *Pf*PFD1-6

*Pf*PFD1-6 were PCR amplified from the cDNA of *Pf*3D7 using gene-specific primers. CDS (coding sequence) encoding for *Pf*PFD1-6 (PF3D7_1107500: *Pf*PFD1, PF3D7_1416900: *Pf*PFD2, PF3D7_0718500: *Pf*PFD3, PF3D7_0904500: *Pf*PFD4, PF3D7_1128100: *Pf*PFD5, and PF3D7_0512000: *Pf*PFD6) were cloned in pET-28a(+) vector (Novagen, Merck KGaA) at BamHI and XhoI restriction sites, and over-expressed in *E. coli* BL21 (ʎDE3). Ni-NTA (Qiagen) affinity purification of *Pf*PFD1-6 was done in lysis buffer (50 mM Tris/HCl, 300 mM NaCl, and 0.02% Na-azide, pH 8.0).

### Generation of antisera against *Pf*PFDs

To raise antibodies against *Pf*PFD1-6, three male BalB/c mice (6–8 weeks old) were each administered (i.p.) with 50 μg of the recombinant *Pf*PFDs (in 0.9% saline), in a prime and boost regimen. A formulation for the priming dose (day 0) was prepared by thoroughly combining equal parts of Freund's complete adjuvant and saline containing the protein. Freund's incomplete adjuvant was used to make formulations for the following booster doses (days 21 and 42). After primary immunization, blood samples were collected from the retro-orbital sinus of the mice on days 31 and 52 (terminal bleed). Extracted blood samples were incubated at 37 °C for 30 min before being centrifuged at 1,200*g* for 15 min at 4 °C, and serum samples were collected and stored at −80 °C until further analysis. The raised antisera were checked for specificity by performing western blotting.

### Co-immunoprecipitation assay to confirm the interaction of *Pf*K13 with *Pf*PFD6, and *Pf*PFD with other subunits and their substrates

Co-immunoprecipitation assay was performed using a PierceTM Co-Immunoprecipitation (Co-IP) kit to confirm the interaction of *Pf*K13 with *Pf*PFD6. Briefly, the anti-*Pf*PFD6 antibody was cross-linked to AminoLink plus coupling beads. After extensive washing, beads were incubated with mixed-stage *Pf*3D7 and *Pf*Kelch13^R539T^ parasite lysate. Parasite lysate was generated by subjecting the mixed-stage culture (∼8% parasitemia) to 0.15% saponin lysis, followed by RIPA lysis of the purified parasites. Bound protein was eluted in the elution buffer, separated on 12% SDS-PAGE, and subjected to western blotting. The blot was probed with a polyclonal anti-K13 antibody (1:1000) followed by a secondary anti-rat (1:5000) antibody. The blot was developed by using the ECL substrate.

A similar protocol was followed to confirm the assembly of the *Pf*PFD complex and its interaction with specific protein substrate α-tubulin-I. To evaluate *Pf*PFD complex formation, *Pf*PFD6 antisera was cross-linked to AminoLink plus coupling beads followed by extensive washing with wash buffer. Mixed-stage culture (∼8% parasitemia) was subjected to saponin lysis, followed by RIPA lysis of the purified parasites. Beads cross-linked with the antisera were incubated with the parasite lysate overnight at 4 °C. Elutes were collected, divided into six groups, and resolved (along with appropriate control, r*Pf*PFDs) on 12% SDS-PAGE, and transferred to nitrocellulose membrane. Blots were individually probed with *Pf*PFD1-6 antisera (1:1000 of each) and secondary HRP conjugated anti-mice antibodies (1:5000; Sigma Aldrich), and developed using diaminobenzidine/H_2_O_2_ substrate (Sigma-Aldrich). To test *Pf*PFD2-α-tubulin-I interaction, anti *Pf*PFD2 antibodies were crosslinked and incubated with parasite lystae. Western blotting was performed by probing with anti-α-tubulin-I antibodies (1:1000). Reverse Co-IP was carried out by crosslinking α-tubulin-I antibody and eluted fractions were probed with anti-*Pf*PFD2 antibodies (1:1000).

### Real-time PCR analysis of *Pf*PFD1-6

Expression of *Pf*PFD1-6 at transcript levels was evaluated in intra-erythrocytic stages of *Pf*3D7 using Real-Time PCR (StepOnePlusTM Real-Time PCR system, Applied Biosystems). 18S rRNA served as a positive control. The primer sequences for the real-time PCR analysis of *Pf*PFD1-6 and 18S rRNA are mentioned in Table S1. The reaction mixture (10 μl) consists of cDNA, SYBR Green PCR Master Mix (5 μl), and *Pf*PFD1-6 specific forward and reverse primers (5 μM). Initial denaturation in PCR was set at 95 °C for 5 min, followed by amplification for 40 cycles of 15 s each at 95 °C, 5 s at 55 °C, and 1 min at 72 °C. Post this, amplification was performed by a melt program comprising of 15 s at 95 °C, 1 min at 60 °C, and a stepwise temperature increment of 0.3 °C/s until 95 °C. Fluorescence acquisition was carried out at each temperature transition. All samples were analyzed in duplicates.

### Expression analysis of *Pf*PFD1-6 by immunoblotting

Mixed-stage asexual cultures of *Pf*3D7 (5–10% parasitemia) were subjected to saponin lysis, followed by RIPA lysis of the purified parasites. The parasite lysate (10 μg of total protein), recombinant proteins (positive control), the hemoglobin from the cytosolic fraction of parasitized RBCs, unparasitized RBCs, and crude extract of *E. coli* (negative control) were resolved on 12% SDS-PAGE and transferred to nitrocellulose membrane. The transferred blots were blocked with 5% BSA in PBS overnight at 4 °C, and probed with mice *Pf*PFD1-6 antisera (1:5000 of each) followed by incubation with Horseradish Peroxidase (HRP)-conjugated goat anti-mice IgG (1:2000). Blots were developed by using diaminobenzidine/H_2_O_2_ substrate (Sigma-Aldrich).

To check for the stage-specific expression of *Pf*PFD1-6, synchronized ring, trophozoite, and schizont stages of the parasite were harvested separately and subjected to saponin lysis. Parasite pellets, thus obtained, were lysed with RIPA buffer for 30 min at 4 °C to rupture the parasite’s membrane and release its cytosolic content. Supernatants of the parasite lysate (10 μg of the total protein) prepared for all three stages were resolved on 12% SDS-PAGE and transferred to the nitrocellulose membrane. The blot was blocked with 5% BSA in PBS overnight at 4 °C, and probed with *Pf*PFD1-6 antisera (1:5000 of each) followed by incubation with HRP-conjugated goat anti-mice IgG (1:2000). Blots were developed using diaminobenzidine/H_2_O_2_ substrate (Sigma-Aldrich).

A similar protocol was followed to check the expression of *Pf*PFDs in *P. falciparum* 3D7 sensitive and ART-resistant *Pf*Kelch13^R539T^ parasites. Synchronized ring, trophozoite, and schizont stage parasite lysate were prepared in RIPA buffer, and an equal amount of protein was loaded on 12% SDS-PAGE and transferred to the nitrocellulose membrane. The membrane was blocked in 5% skimmed milk, followed by probing with primary PFDs anti-sera (1:1000) and secondary anti-mice (1:1000) antibodies.

### Immunofluorescence assays

Thin smears of mixed-stage parasite culture were fixed in ice-cold methanol for 30 min at −20ºC. Fixed smears were permeabilized with PBS/Tween-20, and blocked with 5% BSA (w/v) in PBS for 2 h at RT. For localization studies, *Pf*PFD1-6 antisera (1:200 of each) were added followed by incubation at RT for 2 h. Alexa Fluor 488 conjugated anti-mice (1:250; green; Molecular Probes, Invitrogen) was used as a secondary antibody. For co-localization studies, anti-mice *Pf*PFD1-6 (1:200 of each), anti-rabbit *Pf*NapL (1:250), and anti-rabbit *Pf*MSP1 (1:250) were used. Alexa Fluor 488 conjugated anti-mice and Alexa Fluor 546 conjugated anti-rabbit (1:250; red; Molecular Probes) were used as secondary antibodies. DAPI-antifade (Invitrogen, Life Technologies corporation) was used to counterstain parasite nuclei followed by mounting the slides with coverslips. The slides were viewed under a confocal microscope at 100× magnification (Olympus Corporation).

### MicroScale thermophoresis assays

Binding affinities among the *Pf*PFD subunits were evaluated by MST analyses, using Monolith NT.115 instrument (NanoTemper Technologies). MST detects binding-induced changes in the thermophoretic mobility of a macromolecule, influenced by factors like particle charge, size, conformation, hydration state, and solvation entropy. Under the same buffer conditions, unbound proteins displayed different thermophoresis compared to proteins bound to their interacting partners. In this experiment, 20 μM of *Pf*PFD3 and *Pf*PFD5 were labeled using NanoTemper’s Protein Labelling Kit RED-NHS (L001, NanoTemper technologies) (25). These labeled proteins were titrated with decreasing concentrations of other *Pf*PFD subunits in 1× PBS (pH 7.5) containing 0.01% tween-20. Samples were pre-mixed and incubated for 10 min at room temperature in the dark, then loaded into standard treated capillaries (K002 Monolith NT.115). Interaction analysis measured changes in thermophoresis which is reflected in fluorescence change in the MST signal and is defined as Fhot/Fcold (Fhot being the hot region after IR laser heating and Fcold the cold region at 0 s). Titration of the non-labeled ligand resulted in a gradual change in thermophoresis and is plotted as ΔFnorm to produce a dose-response curve. This dose–response curve is fitted to estimate binding constants. Data analysis was performed using Monolith software (Nano Temper).

Competitive MST analysis was done to check whether BPD hinders the interaction among the PFD subunits. Towards this, the labelled *Pf*PFD3 was mixed with *Pf*PFD1 (7.5 uM), and incubated for 10 to 15 min. The *Pf*PFD3-*Pf*PFD1 complexes, thus formed, were titrated with serial dilutions of BPD in PBS (with 0.01% tween-20) starting from 100 μM, and the interaction analysis was done under the same conditions as described above.

### Generating the 3D-structure model of the *Pf*PFD complex

Amino acid sequences of the PFD subunits 1 to 6 from *P. falciparum* strain 3D7 (1: PF3D7_1107500; 2: PF3D7_1416900, 3: PF3D7_0718500, 4: PF3D7_0904500, 5: PF3D7_1128100, and 6: PF3D7_0512000) were retrieved from the PlasmoDB database (https://plasmodb.org/plasmo/app) (26). A multiple threading approach, which is one of the most common structure prediction methods in structural genomics and proteomics, was employed to generate 3D-structural coordinates of the *Pf*PFD subunits. To accomplish this feat, individual structural models of each subunit were generated using I-TASSER (Iterative Threading ASSEmbly Refinement), a web server that uses a hierarchical approach to protein structure prediction and structure-based function annotation (https://zhanggroup.org/I-TASSER/) (27), as described previously (28). Structural models of *Pf*PFD subunits 1 to 6, thus generated, with higher values of Confidence-score (C-score) were selected and subjected to structural refinement by using ModRefiner (https://zhanglab. ccmb.med.umich.edu/ModRefiner/) which is an algorithm-based approach for atomic-level, high-resolution protein structure refinement (29). The refined structural models of *Pf*PFD subunits were rendered with PyMOL Molecular Graphics System, v2.1 by Schrödinger, LLC (http://pymol.org/2/) (30), and set to submit to generate *Pf*PFDhexamer structure.

The X-Ray diffraction-based structural model of the human TRiC (T-complex protein Ring Complex, also known as Chaperonin Containing TCP-1 (CCT))-PFD complex (PDB ID: 6NR8; resolution: 7.80 Å) (11) was used as a suitable template to generate a 3D structural model of the *Pf*PFDhexamer, as described previously (31). The reliability of the *Pf*PFD structural model was assessed by examining backbone dihedral (torsion) angles: phi (Ø) and psi (Ψ) of the amino acid residues lying in the energetically favourable regions of Ramachandran space (32). This was done by using PROCHECK v.3.5 which checks the stereochemical quality of a protein structure, producing several PostScript plots analyzing its overall and residue-by-residue geometry (https://www.ebi.ac.uk/thornton-srv/software/PROCHECK/) (33). Percent quality measurement of the protein structures was evaluated by using four sorts of occupancies called “core,” “additional allowed,” “generously allowed,” and “disallowed” regions. The 3D structural model of *Pf*PFD, thus generated, was subsequently used for *in silico* and *in vitro* interaction analysis, and inhibition studies.

### LOPAC1280 library screening for a possible inhibitor of *Pf*PFD-mediated protein folding

In a recent investigation by Aline Bamia *et al.*, novel small molecules with Prion (PrPSc) propagation-inhibitory activities were identified, which interfered with the Protein Folding Activity of the Ribosome (PFAR), and significantly prolonged the survival of prion-infected mice (13). Using an *in silico* therapeutic repositioning approach, we screened LOPAC1280 Library of 1280 Pharmacologically Active Compounds (Sigma-Aldrich) based on similarities with one of the potent PFAR inhibitors identified in the study, Metixene (https://www.sigmaaldrich.com/IN/en/product/sigma/lo1280). Metixene is a member of piperidines and has a role as an antiparkinson drug and a muscarinic antagonist. Structural Data Format (SDF) files of Metixene and LOPAC1280 library were retrieved from PubChem, a database of freely accessible chemical information of chemical molecules and their activities against biological assays (https://pubchem.ncbi.nlm.nih.gov/), and Sigma-Aldrich, respectively. Structural superimposition of LOPAC1280 ligands with Metixene was done by using Discovery Studio Visualizer v20.1.0.19295, developed by Dassault Systèms Biovia Corp. (https://www.3ds.com/products-services/biovia/), and overlay structural similarity for each of the 1280 compounds were evaluated, taking Metixene as a reference compound. Structural superimposition analysis was done by using ChemDraw Ultra v12.0.2.1076, one of the CambridgeSoft products for producing a nearly unlimited variety of biological and chemical drawings (https://perkinelmerinformatics.com/products/research/chemdraw).

### *In silico* interaction analysis of BPD with *Pf*PFD

Structural Data Format (SDF) file of BPD.HCl was retrieved from PubChem, and converted to standard PDB format, followed by the generation of its energy-minimized 3D-structural model by using Chem3D Pro 12.0, as described previously (28, 34). Molecular docking studies were performed by using Autodock Vina Tools 1.5.6 to rationalize the inhibitory activity of BPD against *Pf*PFD (28, 34, 35, 36, 37). We ensured that the entire *Pf*PFD complex was covered while constructing a virtual 3D grid for the *in silico* interaction analysis. A grid of 80 × 100 × 80 with x, y, and z coordinates of the center of energy, 209.514, 147.278, and 209.119, respectively was constructed through the Autogrid module of AutoDock Tools, with default spacing. Top scoring docked conformations of BPD were selected based on their most negative free binding energies and visualized for polar contacts (H-bonds; if any) with the amino acid residues of *Pf*PFD complex using PyMOL Molecular Graphics System (30).

### Parasite growth inhibition assay

To evaluate the effect of BPD on the intra-erythrocytic proliferation of the parasite, a growth inhibition assay was performed. Briefly, *Pf*3D7 culture synchronized at ring stage (0.8% parasitemia and 2% hematocrit) was treated with BPD (250 nM, 500 nM, 750 nM, 1 μM, 2.5 μM, and 5 μM). Dilutions of BPD were prepared in iRPMI (Incomplete RPMI; (GibcoTM) medium supplemented with 5.9 gm/L HEPES (Sigma-Aldrich), 50 mg/L hypoxanthine (Sigma-Aldrich), 2 gm/L sodium bicarbonate (Sigma-Aldrich), and 10 mg/L Gentamicin (Sigma-Aldrich). The assay plate was maintained at 37 °C in a controlled atmosphere (5% O_2_, 5% CO_2_, and 90% N_2_) for 72 h. Post-incubation, Giemsa-stained thin blood smears of *P. falciparum* were prepared and ∼3000 RBCs were counted in each smear. The experiment was performed in triplicates. The percentage of growth inhibition was calculated by using the formula: % Inhibition = [1 - % Parasitemia treatment/% Parasitemia control] ∗ 100. The graph was plotted using GraphPad PRISM software.

### Enzyme-linked immune sorbent assay (ELISA) to evaluate the interaction of *Pf*PFD1-6 with α-tubulin-I

To assess the interaction of *Pf*PFD1-6 with α-tubulin-I, a 96-well ELISA plate was coated with purified *Pf*PFD1-6 (bait; 100 ng of each) in PBS at RT for 5 h, and blocked overnight at 4°C with 5% BSA in PBS, followed by incubation at RT for 2 h with increasing concentrations (0–100 ng) of α-tubulin-I (prey). After washing with PBS, antisera (1:10,000) against the respective *Pf*PFD subunits was added to each well, followed by incubation with secondary anti-mice HRP conjugated antibody at RT for 2 h. After washing with PBS, a detection reagent (TMB; HIMEDIA) was added, followed by the addition of 3M HCl to stop the HRP reaction. Absorbance was measured in a microplate reader at 450 nm. The graph was plotted using GraphPad PRISM software.

### Effect of BPD on the expression levels of *Pf*PFD substrates

To evaluate the effect of BPD on the expression levels of α-tubulin-I, trophozoite-parasitized RBCs were treated with BPD (1 and 5 μM). After 6 h of treatment, parasitized erythrocytes were harvested and lysed with 0.05% saponin. The purified parasites, thus obtained, were lysed with RIPA buffer. The parasite lysate was used for western blotting with α-tubulin-I anti-serum to check for the expression levels. Similarly, to assess the effect of BPD on the expression levels of *Pf*MSP-1, trophozoite-parasitized RBCs (at 0.8% parasitemia and 3% hematocrit) were treated with BPD at a concentration equivalent to its IC_50_ (*i.e.*, 1 μM) for 6 and 12 h; whereas, ring parasitized RBCs were treated with 1 μM BPD for 48 h. Thin smears of the BPD-treated cultures were prepared on glass slides for IFA. Similarly, BPD-treated parasites were harvested for western blotting with *Pf*MSP-1 antiserum to check for the expression levels of *Pf*MSP-1.

### Cytotoxic evaluation of BPD on the parasite

To evaluate the cytotoxic effect of BPD on the parasite, BPD (1 and 5 μM) treated trophozoite-parasitized RBCs were co-stained with Propidium Iodide (PI) and SYTO9 fluorescent dyes. Untreated parasitized RBCs served as a negative control. 6 h post-treatment, cells were washed with iRPMI and stained with PI and SYTO9 (100 μM of each; InvitrogenTM Thermo Fisher Scientific) in a 1:1 ratio. Cells were incubated in the dark at RT for 15 min, washed with iRPMI and transferred onto a glass slide for visualization under an Olympus fluorescence microscope. Parasites were treated with DHA (700 nM) served as positive control.

### Parasite egress and invasion assay *in vitro*

To determine the effect of BPD on the invasion and egress rate of the parasite, mature schizonts (45–47 h post-invasion, hpi) were diluted to ∼4% parasitemia and 2% hematocrit. Parasites were treated with varying concentrations of BPD (5, 2.5, 1.25, and 0.625 μM). Untreated parasites were taken as control. After 8 h of treatment, thin smears were prepared on glass slides and stained with Giemsa. Approximately 3000 RBCs were counted under a light microscope at 100× magnification. Percent egress was calculated by using the formula: (No. of schizonts at 0 h - No. of schizonts in treated sample)/(No. of schizonts at 0 h - No. of schizonts in untreated sample) ∗ 100. The initial number of schizonts was taken as 100%. Percent egress inhibition was calculated as 100 percent egress. The number of rings formed per schizont egress: No. of rings/(No. of schizonts pre-treatment - No. of schizonts post-treatment) (38, 39).

### Generation of *Pf*PFD6 complementation strain in yeast mutants

Full-length sequence of *Pf*PFD6 (1–360 bp) was amplified using *P. falciparum* cDNA as a template, and gene-specific primers. The purified insert and p416-GPD vector, with a host range in bacteria and yeast, harbors GPD promotor and bears the Uracil-encoding gene (ura+) for selection, were digested with BamHI/SalI (New England Biolabs) and ligated overnight at 4 °C using T4 DNA ligase (New England Biolabs). The ligation mix was transformed into *E. coli* DH5-α competent cells and positive clones were screened by colony PCR.

The *Pf*PFD6-p416-GPD and p416-GPD constructs were transformed in *S. cerevisiae* mutants YTM1304::pfd6Δ (gim1Δ), using Frozen-EZ Yeast Transformation IITM kit (Zymo Research) as per the manufacturer's protocol and plated on YNB (yeast nitrogen base) agar plate (supplemented with 2% glucose and 1× amino acid mix without uracil) as selective media. Cells were allowed to grow at 30 °C. The transformed colonies were confirmed by colony PCR using gene-specific primers. *S. cerevisiae* BY4742 (MATα; his3Δ; leu2Δ; lys2Δ; ura3Δ) was used as the wild-type control wherever applicable. YTM1304-p416-GPD was also used as a control in experiments.

### Assessment of biperiden selectivity for *Pf*PFD6

To confirm the selectivity of BPD towards *Pf*PFDs, YTM 1304-p416-GPD and YTM 1304-*Pf*pfd6 were cultured in their respective media overnight until sufficient growth was achieved. Subsequently, the cultures were diluted to an optical density, A_600_ of 0.1 using sterile fresh media and further incubated in the absence and presence of 20 μM BPD at 30 °C for 12 h. The resulting cell suspension was then serially diluted to 10-folds, and each dilution was spotted onto a YPD agar plate. The culture plate was incubated for 2 days at 30 °C.

### Assessment of artemisinin effect on growth of complemented mutant yeast strains

To elucidate the impact of ART treatment on the yeast cell growth pattern, YTM1304-*Pf*PFD6, and YTM1304-p416-GPD were grown in YPD media and used as a pre-culture. Once the culture reached appropriate growth, it was diluted to an initial A_600_ value of 0.1 and subjected to further incubation at 30 °C for 12 h, with and without the addition of artemisinin (8 μM). Post-incubation cultures were harvested and adjusted to an A_600_ value of 0.1. The cultures were then subjected to ten-fold serial dilution, spotted on the YPD agar plate, and incubated at 30 °C for the next 2 days.

### Expression analysis of *Pf*PFD6 in YTM 1304 mutants of *S. cerevisiae*

Immunofluorescence assays were performed to check the expression of *PfPFD6 in* YTM 1304 mutants of *S. cerevisiae*. 5 ml of secondary yeast culture with O.D 0.5 to 0.7 was fixed with 37% formaldehyde and incubated for 30 min at 30 °C with intermitted tapping. Cells were then pelleted and washed thrice with 1X PBS and resuspended in 25 μl of 1M DTT and again incubated for 20 min at 30 °C. The cell wall was digested using zymolase (10 mg/ml) and 5ul β-mercaptoethanol, further, the suspension was incubated at 30 °C for 20 min. The culture was pelleted and washed twice with 1XPBS. 0.1% Triton X-100 was used for cell permeabilization and incubated at RT for 20 min. Cells were then blocked using a blocking solution (5 mg/ml BSA+22.5 mg/ml glycine in 1X PBS) and incubated for 10 min at RT. Yeast cells were then pelleted and incubated for 1 h with anti-mice *Pf*PFD6 antisera (1:300) followed by three PBST washes. Post-washing, the cells were incubated for 45 min with anti-mice Alexa fluor-488 antibody (1:300) followed by PBST washes to remove unbound antigens. Yeast pellets were resuspended in 100 μl of 1× PBS and 3 μl of yeast cells were then spotted onto the microscope slide along with DAP1 and a coverslip was applied. Imaging was done using an Olympus fluorescence microscope at 100× magnification.

### Ring survival assays

To evaluate the effect of Biperiden in *Pf*Kelch13 ^R539T^ ART-resistance parasites, a ring survival assay was performed as previously described (22, 40). Briefly, Percoll purified early ring stage parasite (0–3-h ring post-invasion) was treated with DHA (700 nM) for 6 h. Post-washing with iRPMI, the parasite was treated with BPD (1 μM), and incubated at 37º C for 66 h. The parasitemia was calculated by counting the number of ∼3000 parasitized cells. Percent survival was calculated using the following formula:

%survival=100−%Inhibition

%Inhibition=[(C−T/C)]100

C: Parasitaemia of untreated *Pf*Kelch13 R539T, T: Parasitaemia of either DHA treated or DHA plus BPD treated parasites.

### Parasite growth inhibition *in vivo*

BALB/c female mice (6 weeks old) were divided into five groups and each group consisted of four animals. At day 0, mice were infected intra-peritoneally with 1 × 10^6^ infected RBCs (100 μl, diluted in PBS), obtained from *P. berghei* ANKA-infected donor mice. Group 1 mice were treated with artesunate at a concentration of 6 mg/kg (positive control), Group 2 was treated with 12.5 mg/kg of BPD, while Group 3 was treated with a combination of artesunate (6 mg/kg) and BPD (12.5 mg/kg). Group 4 was left untreated (negative control), while Group 5 was not infected by *P. berghei* ANKA. Blood samples from the tail end of the infected mice were taken daily. Percent parasitemia was determined by observing the Giemsa-stained smears of the blood samples under a microscope at 100× magnification (Olympus Corporation).

### Statistical analysis

All graphs were generated by the software GraphPad Prism (version 8) and statistical analysis was calculated by using unpaired two-tailed Student’s t-tests. *p* value ≤ 0.05 was considered significant.

### Ethics approval

The mice-based studies were conducted at Animal House, Jawaharlal Nehru University and were approved by the Institutional Animal Ethics Committee (IAEC) of Jawaharlal Nehru University, New Delhi, India (code no. 35/2019); and, the Committee for the Purpose of Control and Supervision of Experiments on Animals (CPCSEA), Government of India. Mice-based *in vivo* experiments are also reported following the guidelines of “Animal Research: Reporting of *In Vivo* Experiments” (ARRIVE) guidelines (https://arriveguidelines.org/).

## Data availability

Data will be made available on request.

## Supporting information

This article contains supporting information.

## Conflict of interest

The authors declare that they have no conflicts of interest with the contents of this article.

## Supporting information

Table S1

Band 1 mass spec_K-13

Band 2 mass spec_K-13

Band 3 mass spec_K-13

Band 4 mass spec_K-13

Band 5 mass spec_K-13
