# Supplementary material for: Prefoldins are novel regulators of the unfolded protein response in artemisinin resistant Plasmodium falciparum malaria[image]
Source: J Biol Chem. 2024 Jun 24;300(8):107496. doi: 10.1016/j.jbc.2024.107496 (PMC11295463; doi:10.1016/j.jbc.2024.107496)
Supplement: Supplementary file [file mmc1.pdf]

Supporting information

Prefoldins are novel regulators of the unfolded protein response in artemisinin resistant *P. falciparum* malaria

Rumaisha Shoaib[1,2,#], Nidha Parveen[1,#], Vikash Kumar[1,#], Ankita Behl[1], Swati Garg[1], Preeti Chaudhary[3,4], Devasahayam Arokia Balaya Rex[5], Monika Saini[1,6], Preeti Maurya[1], Ravi Jain[1], Kailash C. Pandey[3], Mohammad Abid[2], Shailja Singh[1,*]

[1]Special Centre for Molecular Medicine, Jawaharlal Nehru University, New Delhi, Delhi, 110067; India

[2]Medicinal Chemistry Laboratory, Department of Biosciences, Faculty of Life Sciences, Jamia Millia Islamia, New Delhi, Delhi, 110025; India

[3]Parasite Host Biology Group, ICMR-National Institute of Malaria Research, New Delhi, India

[4]Department of Life Sciences, IGNOU, Delhi, India

[5]Department of Laboratory Medicine and Pathology, Mayo Clinic, Rochester, Minnesota, U.S.

[6]Department of Life Sciences, Shiv Nadar University, Delhi NCR, Uttar Pradesh, 201314 India

[#] Equal contribution

***Corresponding author**

Shailja Singh, Special Centre for Molecular Medicine, Jawaharlal Nehru University, Delhi: 110067, India; email address: shailjasingh@mail.jnu.ac.in; Tel.: 011-26743038; Fax: 011-26742580; **ORCID ID: 0000-0001-5286-6605.**

(A)

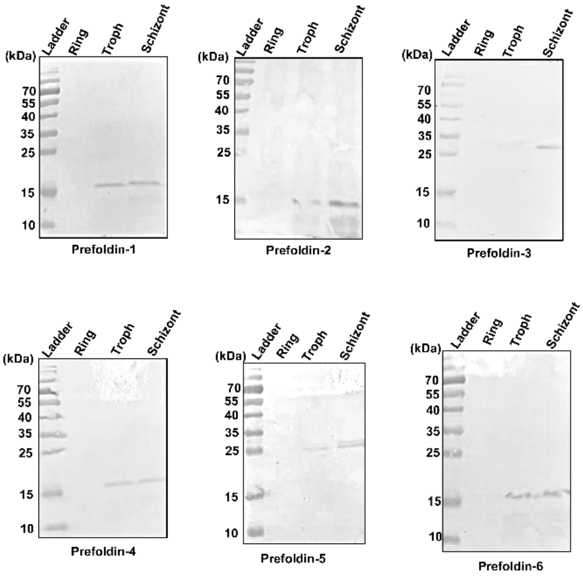

(B)

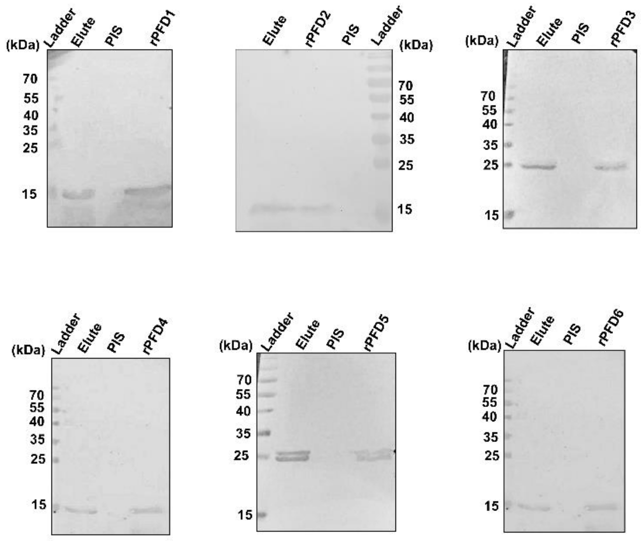

(C)

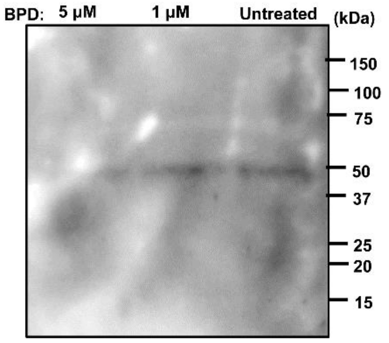

(D)

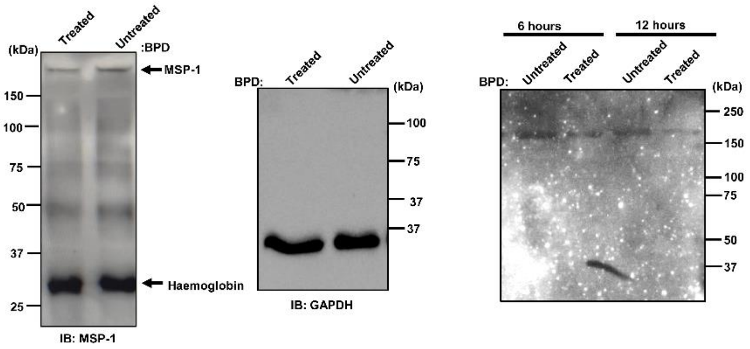

Fig. S2: Images representing the uncropped version of (A) Fig. 2B, (B) Fig. 3B, (C) Fig. 5C, and (D) Fig. 5D (i, iii, & iv) of the main figures.

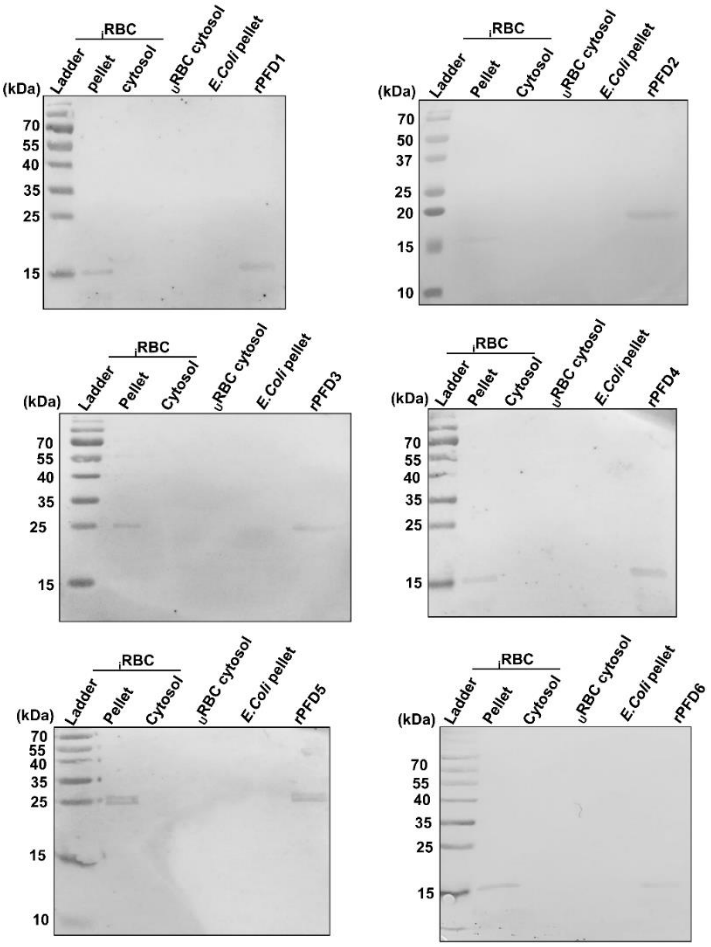

Fig. S3: Evaluation of antibody specificity generated against prefoldin subunits. Western blot analysis of *Pf*PFD1, *Pf*PFD2, *Pf*PFD3, *Pf*PFD4, *Pf*PFD5 and *Pf*PFD 6 in total parasite lysate using their specific antibodies. No band was detected in RBC cytosol and *E. coli* (DH5 alpha) pellet, depicting the specificity of anti-PFDs antibodies.

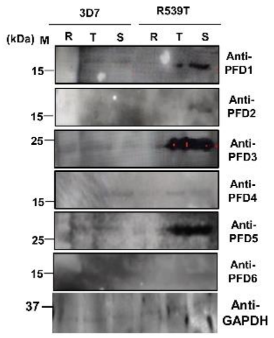

Fig. S4: Upregulated expression of *Pf*PFD1-6 at protein levels in *P. falciparum* R539T. Parasite lysates (*Pf*3D7 and R539T strain) were prepared for different asexual

stages and subjected to western blotting using *Pf*PFD1-6 antisera (1:1,000) and HRP-conjugated anti-mice antibodies (1:5,000).

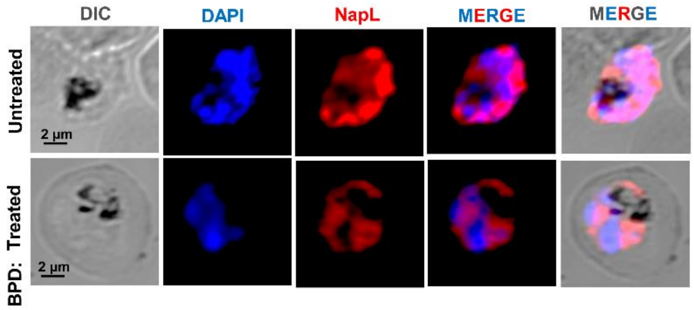

Fig. S5: Effect of BPD on cellular localization of NapL. Methanol-fixed thin blood smears of treated and untreated *Pf*3D7 infected erythrocytes were stained with anti-NapL antisera (1:200) followed by incubation with Alexa Fluor conjugated secondary antibody (1: 200; Alexa Fluor 594, red color). DIC: differential interference contrast image, DAPI: nuclear staining using 4',6-diamidino-2-phenylindole (blue); anti-NapL antibodies (red); merge: overlay of NapL with DAPI.
