# Supplementary material for: Prefoldins are novel regulators of the unfolded protein response in artemisinin resistant Plasmodium falciparum malaria[image]
Source: J Biol Chem. 2024 Jun 24;300(8):107496. doi: 10.1016/j.jbc.2024.107496 (PMC11295463; doi:10.1016/j.jbc.2024.107496)
Supplement: Table S1 [file mmc2.docx]

**Table S1: Primer sequences for the real-time PCR analysis of *Pf*PFD1-6.**

| **Genes** | **Primer sequences (5’-3’)** |
| --- | --- |
| **PFD1** | \| F: TTACAAGATCTTTTTGATATG \| \| --- \| \| R: ATTCCAGTAGGACAATTTCTC \| |
| **PFD2** | \| F: AGAACAGTCGGGGAAATTAAGC \| \| --- \| \| R: TTGTTTTTGACATTCAGCTATAACC \| |
| **PFD3** | \| F: TCTTTGTATGCTAGGGGAATCG \| \| --- \| \| R: AAAGGGAAACTCAACCATTACG \| |
| **PFD4** | \| F: TTAGCTTTTGATTCAAATGAT \| \| --- \| \| R: CTCTTCAGATTTTCTATCCT \| |
| **PFD5** | \| F: TTGAAGAAAATGAAAGAGAAAAACC \| \| --- \| \| R: AAGCCCCAACGTATTCAGC \| |
| **PFD6** | \| F: CTATTTAGCGATTTAGAAGCACACG \| \| --- \| \| R: GGAACGGGTATTTTTCTCTGTCC \| |
| **18s rRNA** | F: 5′- CCGCCCGTCGCTCCTACCG-3′  5′-CCTTGTTACGACTTCTCCTTCC-3′ |
